# Size‐Dependent Ultrafast UV Photochemistry of Aliphatic Disulfides in Solution

**DOI:** 10.1002/chem.202404695

**Published:** 2025-05-26

**Authors:** Jessica Harich, Rory Ma, Miguel Ochmann, Antonia Freibert, Yujin Kim, Minseok Kim, Eunhyo Kim, Madhusudana Gopannagari, Junho Lee, Tae Gyun Woo, Haneol Oh, Ru‐Pan Wang, Jae Hyuk Lee, Tae Kyu Kim, Nils Huse

**Affiliations:** ^1^ Department of Physics University of Hamburg Center for Free-Electron Laser Science Luruper Chaussee 149 22761 Hamburg Germany; ^2^ Pohang Accelerator Laboratory, POSTECH Pohang 37673 Republic of Korea; ^3^ Department of Chemistry Korea Advanced Institute of Science and Technology (KAIST) Daejeon 34141 Republic of Korea

**Keywords:** disulfide, photochemistry, X-ray absorption, geminate recombination, solution

## Abstract

We have investigated the photochemistry of dimethyl disulfide (DMDS) and glutathione disulfide (GSSG) in solution upon ultraviolet (UV) excitation in comparison to L‐cystine by femtosecond X‐ray absorption spectroscopy at the sulfur K‐edge. For these aliphatic disulfides, the very similar differential absorption spectra reveal unequivocally a pair of geminate thiyl radicals as the single primary reaction product with a formation time constant of about 100 fs. We find size‐dependent majority yields for disulfide recombination with DMDS reforming ten‐fold slower and at lower quantum yield than L‐cystine while GSSG recombines even faster than L‐cystine at a yield of nearly 80 % within 2 ps after excitation. We attribute the much lower recombination rates and yields of DMDS to larger product separation after homolytic S−S bond cleavage due to the smaller thiyl radical mass and correspondingly larger mutual acceleration. Furthermore, we observe the delayed formation of at least one additional reaction product for these disulfides that we attribute to perthiyl radicals. The ultrafast recombination of L‐cystine and GSSG at majority yield in condensed phase suggests a dynamic S−S bond integrity which may be exploited in natural systems to mitigate UV radiation damage, and which could be of interest in materials design.

## Graphical abstract

Aliphatic disulfides exclusively split homolytically along the S−S bond when directly exciting the disulfide chromophore at about 250 nm. Ultrafast (<1 ps) geminate recombination at high yield is observed in solution for fragment sizes as small as L‐cysteine. The smallest disulfide recombines ten‐fold more slowly. Ultrafast disulfide reformation will also exist in proteins, providing inherent protection from radiation damage.

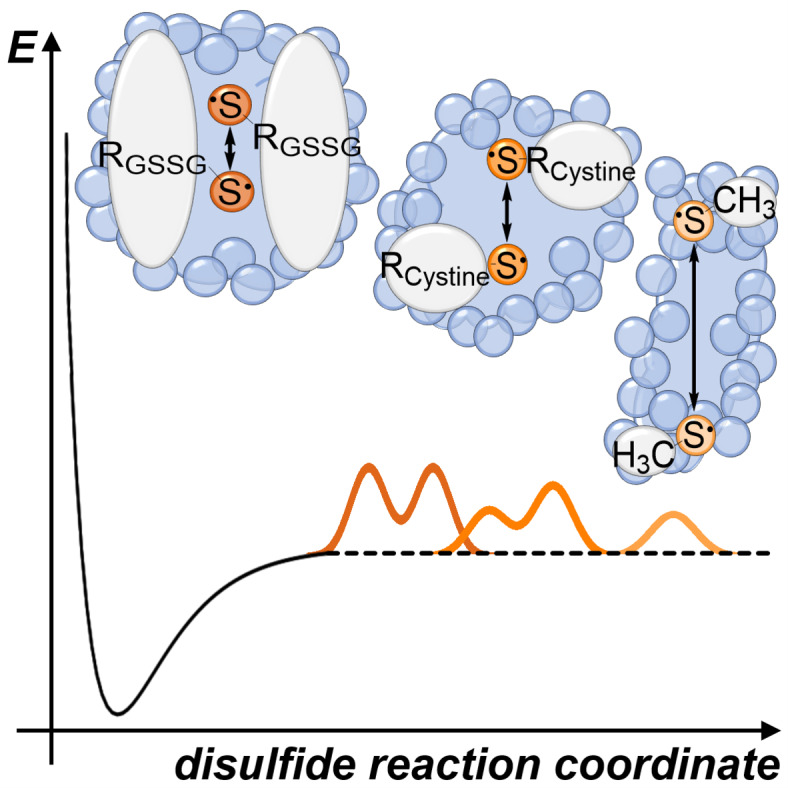

## Introduction

Two sulfur atoms of molecular residues can form a disulfide bond between them with important properties for various scientific fields, including materials science,[^1^, ^2^] chemistry[^3^, ^4^] and life science[^5^, ^6^, ^7^, ^8^, ^9^, ^10^, ^11^]. While dimethyl disulfide (DMDS) as the smallest disulfide is relevant in the global sulfur cycle,[^12^, ^13^] disulfide bonds formed between two cysteine residues are crucial for the structural and functional integrity of proteins where they significantly contribute to the stabilization of the tertiary and quaternary structure of many proteins, thereby maintaining biological function.[^14^, ^15^] In addition to providing structural support, these bonds allow proteins to adapt to cellular stress and environmental changes through their reversible formation and breakage and are thus critical in regulating biological function in many proteins.[^16^, ^17^] Moreover, disulfide bonds can protect the protein from photoinduced chemical or physical degradation. Upon ultraviolet light exposure, the disulfide bond is most prone to break, and does so on a sub‐picosecond timescale,[^18^, ^19^, ^20^] potentially resulting in the loss of protein function and activity.^[21]^ However, the ultrafast cleavage of S−S bonds induced by UV radiation can also serve as a protective mechanism by acting as a radiation shield or a radical scavenger of more reactive species,^[22]^ thereby preventing irreversible damage to proteins. The photodissociation of disulfide bonds and its molecular consequences have been reported for various proteins,[^7^, ^23^, ^24^, ^25^, ^26^, ^27^, ^28^, ^29^, ^30^, ^31^, ^32^, ^33^] highlighting the importance of this mechanism in preserving protein integrity under radiation stress.

To assess, predict, control, or prevent harmful protein photodamage, a mechanistic insight into the early timescales of UV‐induced disulfide bond cleavage and subsequent product formation is therefore of relevance to several scientific disciplines. Indeed, the photochemical behavior of disulfide bonds has been investigated for various aliphatic cyclic[^19^, ^34^, ^35^, ^36^, ^37^] and acyclic model systems,[^38^, ^39^, ^40^, ^41^, ^42^] which serve as simplified representations of the disulfide bridges in proteins. Many static and time‐resolved gas‐phase experiments have been performed on DMDS, a volatile organic compound produced by microbial decomposition of the sulfur‐containing amino acid methionine^[43]^ that is released into the atmosphere as a minority species in the sulfur cycle.^[44]^ These studies have demonstrated a wavelength‐dependent formation of the primary photoproducts. When excited at wavelengths longer than 200 nm, only homolytic S−S bond cleavage occurs, producing thiyl radicals as the primary photoproduct.[^45^, ^46^, ^47^, ^48^] At shorter wavelengths, however, an additional competitive dissociation pathway is observed, leading to perthiyl radicals through C−S bond cleavage.[^49^, ^50^, ^51^, ^52^, ^53^] These findings have been corroborated in other organic disulfides,[^19^, ^29^, ^41^, ^47^, ^54^] underscoring the wavelength‐sensitivity of disulfide photochemistry and its implications for understanding protein photodamage and repair mechanisms.

In contrast to results observed for isolated molecules, C−S bond cleavage has also been detected in solvated disulfides upon irradiation with wavelengths greater than 200 nm.[^42^, ^55^, ^56^] However, the temporal resolution in these experiments was insufficient to elucidate the precise reaction mechanisms. Our recent study^[57]^ on the UV photochemistry of the dianion L‐cystine, using 267 nm excitation, provides new insight by use of femtosecond X‐ray absorption spectroscopy at the sulfur K‐edge. This technique unambiguously showed that the primary photoproduct is a pair of geminate thiyl radicals resulting from homolytic S−S bond cleavage. In contrast, perthiyl radicals, which are products of C−S bond cleavage, may only form as secondary photoproducts through solvent‐mediated recombination where the confining forces of the solvent cage enable geminate recombination and post‐fragmentation reactions, typically inaccessible in the gas phase. Additionally, the solvent environment serves as a medium for dissipation of excess energy after UV irradiation, making it a key ingredient in disulfide photochemistry in condensed phases.

In comparison with cystine and DMDS, the photochemistry of the biologically highly relevant glutathione disulfide (GSSG), the dimer of the tripeptide glutathione (GSH), has not been well studied with time‐resolved methods. Everett et al. noted the production of thiyl radicals upon 254 nm excitation in their study of multiple disulfides and trisulfides in solution,^[58]^ and Sneeden et al. observed the production of thiyl radicals from GSH during the recording of X‐ray absorption spectra at the sulfur K‐edge. The lack of time‐resolved spectroscopic studies of disulfide and thiol photochemistry may surprise, given the importance of GSH and GSSG in various cellular processes and molecular structures. GSH – generally by far the most abundant intracellular non‐protein thiol – is involved in preventing and mediating oxidative and nitrosative cellular damage.[^59^, ^60^] GSSG plays an important role in oxidative protein folding of disulfide containing proteins in a thiol‐disulfide exchange reaction. *S*‐glutathionylation of protein thiols is further associated with critical oxidative signaling events.[^61^, ^62^] Moreover, the important GSH/GSSG ratio within cells is regulated by enzymatic activity and intracellular transport.[^59^, ^61^]

**Figure 1:**
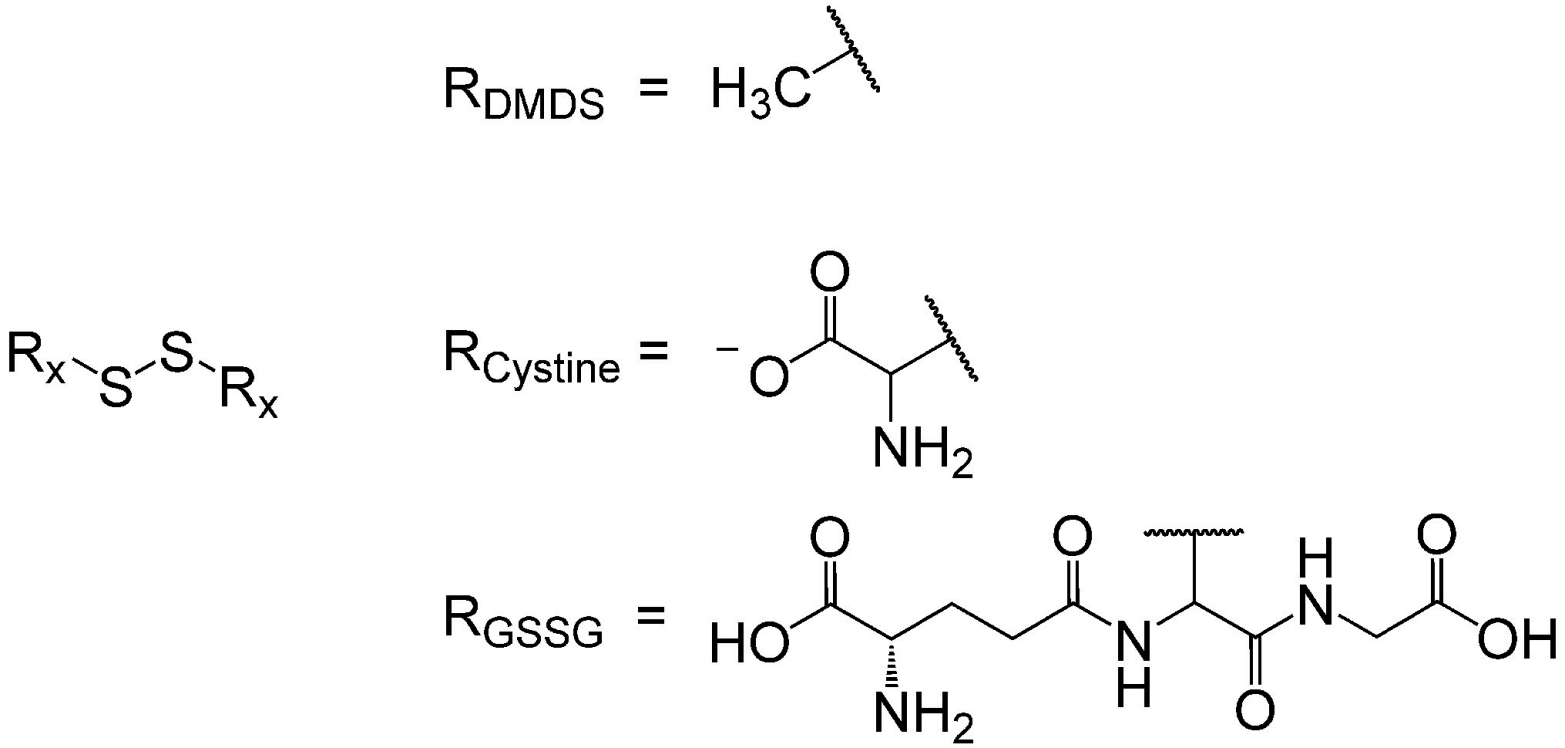
Structural motif of disulfides (R_x_SSR_x_) and the residues distinguishing the three aliphatic disulfides investigated in this study: Dimethyl disulfide (R_DMDS_), the cystine dianion (R_Cystine_) and glutathione disulfide (R_GSSG_).

In this photochemical work, we study the size‐dependent photochemistry of aliphatic disulfides in solution upon UV excitation using time‐resolved X‐ray absorption spectroscopy at the sulfur K‐edge. By comparing the temporal evolution of photoproducts following pulsed UV irradiation at 267 nm for three aliphatic disulfides (Figure 1) – DMDS, the dianion L‐cystine (hereafter simply referred to as ‘cystine’), and GSSG – we provide a better understanding of the early photochemical processes of the disulfide bond motif in molecules of increasing complexity and size within a solvent environment.

## Experimental

### Experimental Setup

The measurements of the three disulfide species were conducted at the NCI beamline of the PAL‐XFEL free electron laser in Pohang, South Korea. Details of the experimental setup have been described elsewhere.[^63^, ^64^, ^65^] Briefly, the XFEL delivered monochromatized X‐ray pulses at a repetition rate of 60 Hz into the sample chamber held at 1 atm of helium. The X‐ray pulses were focused to 14x15 μm^2^ (cystine and DMDS) and 25×25 μm^2^ (GSSG) at the sample position. Our photon energy calibration, takes several X‐ray sources into account (see Supporting Information of Ochmann et al.).^[42]^ The shifts were as follows: DMDS and cystine: +0.3 eV, GSSG: −0.3 eV.

For the X‐ray absorption measurements of the samples, a round steel nozzle of 150 μm diameter (the exit of which was placed 0.5 mm above the X‐ray focus) provided the vertical liquid sample jet which was collected below the X‐ray focus by a stainless steel cylinder connected to PTFE tubing. An avalanche photodiode (APD, Excelitas Technologies C30703FH‐200, 10×10 mm^2^ active area), was placed at right angle to the X‐ray beam and the sample jet to detect the X‐ray fluorescence at a distance of 1 cm from the X‐ray focus. The APD was negatively biased at 130 V and shielded with a 1.6 μm thick aluminum foil. APD signals were amplified by a transimpedance amplifier (DHPCA‐100, FEMTO Messtechnik GmbH) at a gain setting of 1000 V/A in low‐noise mode (80 MHz full bandwidth), yielding signals between 60 mV at 2467 eV (40 mV elastic scattering and electronic background, 20 mV fluorescence by Lorentzian absorption tail) and ~1 V at the absorption maximum.

The pulses of an amplified femtosecond titanium‐sapphire laser system with 30 Hz repetition rate and 100 fs pulse duration (FWHM) were converted to 267 nm pulses by third‐harmonic generation for sample excitation. A maximum power density of 0.75 TW/cm^2^ was used for measurements of differential X‐ray absorption spectra (DMDS: 0.18 TW/cm^2^, 0.25 TW/cm^2^ and 0.50 TW/cm^2^, cystine: 0.75 TW/cm^2^, GSSG: 0.46 TW/cm^2^). For the measurements of cystine and DMDS the diameter of the round laser focus was 145 μm (1/e^2^), for the measurements of GSSG the focal diameter was 107 μm (1/e2). From the pulse width of the two lasers ($\sigma_{UV}$ =45 fs and $\sigma_{X-ray}$ =20 fs), the temporal jitter and drift between the two lasers of 30 fs and the broadening from the sample due to group velocity dispersion of 35 fs to 70 fs, the width of the instrument response function (IRF) $\sigma_{IRF}$ was fit between 70 fs (GSSG) and 90 fs (DMDS, cystine).

The measured X‐ray fluorescence intensity (total fluorescence yield, TFY) is to good approximation proportional to the X‐ray absorption of the sample. Therefore, the change of absorption is proportional to the difference of the TFY spectra of the UV‐excited sample and the unexcited sample (TFY_UV_−TFY_0_).

### Materials

DMDS (≥98 %), L‐cystine (99.7 % TLC), GSSG (≥98 %) and sodium hydroxide (BioXtra, ≥98 %) were purchased from Sigma‐Aldrich South Korea and were used as received without further purification. Solutions of 0.94 g DMDS (100 mM) in 100 mL methylcyclohexane and 6.13 g GSSG (100 mM) in 100 mL MQ water were prepared. In total 200 mL aqueous solution of 4.80 g cystine (100 mM) and 2.39 g NaOH (300 mM) were prepared, which gave two portions of 100 mL sample solution. The 100 mL sample solutions were loaded into the jet‘s sample reservoir for measurements. The solutions were replaced every 5 hours when running in a continuous loop. No change in the static X‐ray absorption spectra was observed during the measurements.

### Kinetic Modelling

For each of the three investigated disulfides transient delay scans at three characteristic spectral positions have been modelled by the solution of a rate‐equation model convolved with a Gaussian instrument response function, that has been further developed from the model originally developed for cystine and discussed in detail in a previous publication.^[57]^ Briefly, the $N_{ij}\left(t\right)\;$ are time‐dependent partial populations: $N_{1}$ is the excited‐state population, $N_{2}$ is the primary photoproduct's population, $N_{3}$ is the population of the vibrationally excited state (VES) of the recombined parent molecule, $N_{4}$ is the secondary photoproduct's population and $N_{0}$ is the population of the parent molecule. To account for the observed multiple relaxation timescales, the populations of $N_{2}$ , $N_{3}$ , and $N_{4}$ are sums of statistical sub‐ensembles. The rate constants of the population decays are $k_{1}=1/\tau_{1}$ and $k_{ij}=1/\tau_{ij}$ .

(1) $${\dot{N}}_{1}\left(t\right)=-\;k_{1}N_{1}\left(t\right)$$

(2) $${\dot{N}}_{2i}\left(t\right)=k_{1}N_{1}\left(t\right)-k_{2i}N_{2i}\left(t\right)$$

(3) $${\dot{N}}_{3j}^{2i}\left(t\right)=k_{2i}N_{2i}\left(t\right)-k_{3}N_{3j}^{2i}\left(t\right)$$

(4) $${\dot{N}}_{4j}^{2i}\left(t\right)=k_{3}N_{32}^{2i}\left(t\right)-k_{4j}N_{4j}^{2i}\left(t\right)$$

(5) $${\dot{N}}_{03}^{2i}\left(t\right)=k_{3}N_{31}^{2i}\left(t\right),\;{\dot{N}}_{04j}^{2i}\left(t\right)=k_{4}N_{4j}^{2i}\left(t\right)$$

The relative yields $\varphi_{ij}$ of the sub‐ensembles are defined by the sum of ensemble populations:

(6) $$N_{2}=\sum_{i=1}^{3}\varphi_{2i}\;N_{2i}$$

(7) $$N_{3}=\sum_{j=1}^{2}\varphi_{3j}\;N_{3j}=\sum_{j=1}^{2}\varphi_{3j}\sum_{i=1}^{3}\varphi_{2i}\;N_{3j}^{2i}$$

(8) $$N_{4}=\sum_{j=1}^{2}\varphi_{4j}\;N_{4j}=\sum_{j=1}^{2}\varphi_{4j}\;\sum_{i=1}^{3}\varphi_{32}\;\varphi_{2i}\;N_{4j}^{2i}$$

(9) $$N_{0}=\sum_{i=1}^{3}\varphi_{31}\;\varphi_{2i}\;N_{03}^{2i}+\sum_{j=1}^{2}\varphi_{4j}\sum_{i=1}^{3}\varphi_{32}\;\varphi_{2i}\;N_{04j}^{2i}$$

with $N_{1}\left(0\right)=1$ and $N_{2}\left(0\right)=N_{3}\left(0\right)=N_{4}\left(0\right)=N_{0}\left(0\right)$ =0. Yields sum according to $\sum_{i=1}^{3}\varphi_{2i}=\sum_{j=1}^{2}\varphi_{3j}=\sum_{j=1}^{2}\varphi_{4j}$ =1. For DMDS, we have set $\varphi_{21}=0$ because no ultrafast (<1 ps) relaxation is observed. We have also set $\varphi_{41}=1$ for DMDS and GSSG because no biexponential decay of the secondary product is discernible over the measured time delays.

The solution of the differential rate equation model is given in the Supporting Information (SI) alongside a schematic representation of its kinetic pathways. Within our model, the antibonding excited state $N_{1}$ is not observable. $N_{1}$ is assumed to feed into the first observable product state, $N_{2}$ , which then feeds into $N_{3}$ . It is reasonable to assume that $N_{3}$ does not accumulate observable population and feeds into either the repopulation of the parent state $N_{0}$ or the secondary product state $N_{4}$ . Assuming a Gaussian shape, the widths of the instrument response functions (IRF) were fit to $\sigma_{IRF,\;DMDS}=\left(0.090\pm 0.007\right)$ ps, $\sigma_{IRF,Cystine}=\left(0.098\pm 0.006\right)$ ps and $\sigma_{IRF,GSSG}=\left(0.068\pm 0.007\right)$ ps, equivalent to an experimental time resolution of 150 fs to 250 fs full width at half maximum (FWHM). The modeling was done with global time constants $\tau_{1}$ and $\tau_{ij}$ by fitting them simultaneously to all three transients of each disulfide.

## Results and Discussion

### X‐Ray Absorption Spectra

**Figure 2:**
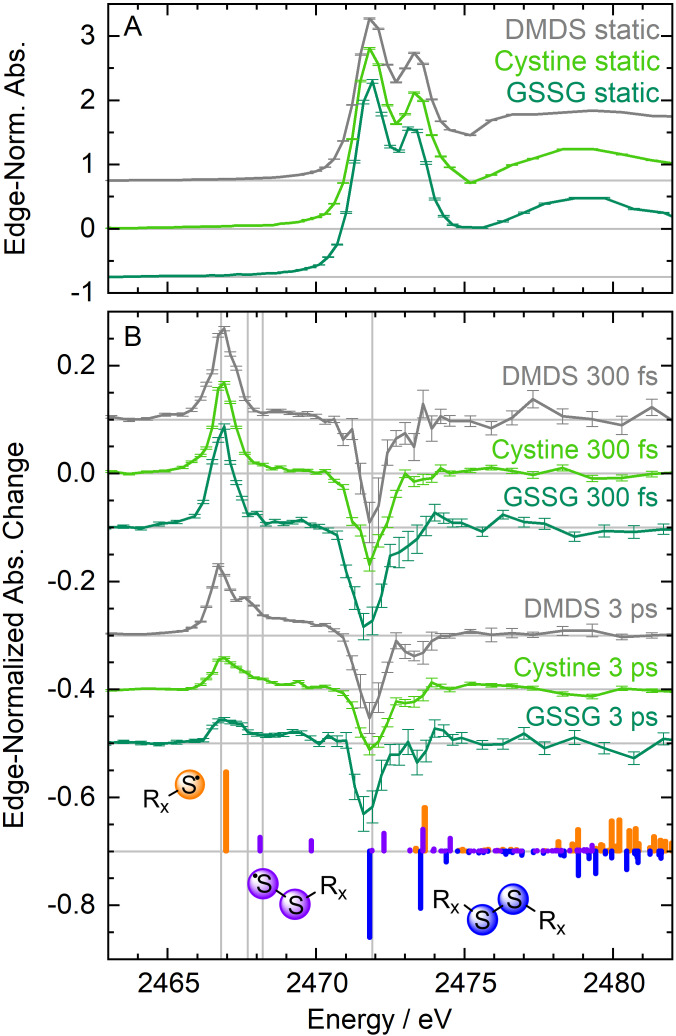
Static and differential sulfur K‐edge absorption spectra of dimethyl disulfide (DMDS, grey), cystine (light green) and glutathione disulfide (GSSG, dark green). All spectra and theory calculations of cystine are taken from a previous publication.^[57]^ **A**) Static spectra of dimethyl disulfide (offset by +0.75), cystine, and glutathione disulfide (offset by −0.75). **B**) Differential spectra at 300 fs and 3 ps temporal delay after 267 nm excitation of DMDS (300 fs offset by +0.1, 3 ps offset by −0.3), cystine (3 ps offset by −0.4) and GSSG (300 fs offset by −0.1, 3 ps offset by −0.5). The absorption changes of cystine are normalized to its K‐edge height. Absorption changes of DMDS and GSSG are scaled relative to the absorption change of cystine at 2466.8 eV. Vertical grey lines indicate the probe energies at which delay scans were recorded. Exemplary calculated transitions for cystine (blue), the cysteinylthiyl radical (orange) and for the cysteinylperthiyl radical (purple) are shown.

Static absorption spectra of DMDS, cystine and GSSG (measured by total fluorescence yield) are shown in Figure 2A. The spectra of DMDS and GSSG have been offset for better visibility. The three spectra are very similar in shape with two clearly discernable lowest transitions at near 2472 eV and 2473.5 eV. The absorption spectra have been normalized to the sulfur K‐edge. A lineshape analysis of the DMDS and GSSG spectra reveals inhomogeneous broadening of 0.8 eV (c.f. SI), similar to cystine^[57]^ and attributable to a distribution of thermally accessible disulfide conformers at room temperature in solution. A recent study finds spectral shifts similar to this broadening in sulfur‐2p photoelectron spectra of disulfides with different dihedral angles.^[66]^

Differential absorption spectra of the three investigated molecules at time delays of 0.3 ps and 3 ps after UV excitation are plotted in Figure 2B. Again, the spectral shapes are very similar, showing decreases where absorption is lost and increases where absorption is gained. 300 fs after 267 nm excitation a (positive) single homogeneously broadened Lorentzian lineshape at 2466.8 eV and a (negative) bleach signal at 2471.8 eV is observed for all three disulfides (Figure 2B). For comparison, the difference spectra have been scaled to the induced absorption peak at 2466.8 eV of the cystine difference spectrum, which in turn is given as an absorption change relative to the sulfur K‐edge height of the static spectrum (the latter is normalized to 1). The homogeneity of the lineshape at 2466.8 eV clearly suggests a single product and no conformer distribution near the sulfur atoms.

At a delay of 3 ps the similarity between the time‐resolved differential spectra remains. However, the peak at 2466.8 eV has decayed and is less pronounced. A set of transitions manifests as a shoulder between 2468 eV and 2471 eV. Compared to the very similar spectra of cystine and GSSG after 3 ps the difference spectrum of DMDS deviates somewhat: The absorption peak at 2466.8 V and the bleach signal at 2471.8 eV have relaxed less and are most pronounced. Additionally, the shoulder that has appeared as a secondary feature, is stronger than the shoulder developed in either cystine or GSSG.

We modeled the time‐resolved differential spectra of DMDS, cystine and GSSG by calculating the lowest sulfur‐1s transitions with time‐dependent density functional theory (TD‐DFT) at the same level as was previously done for cystine (see also Figure S1 of SI). We find very good agreement with experimental bleaching and induced absorption signals: For the parent molecules, excitations of sulfur‐1s electrons to sulfur‐based π*‐orbitals characterize the transitions of the static spectrum and the bleaching signal. The induced absorption lineshape at 2466.8 eV 300 fs after 267 nm excitation is identified as the primary and sole photoproduct: The two (identical) thiyl radicals, produced upon S−S homolytic bond cleavage. Due to the strong similarity of the DMDS and GSSG difference spectra with those of cystine at 300 fs delay, we conclude that the initial reaction pathway upon 267 nm excitation is the same in the three aliphatic disulfides, yielding the methylthiyl and glutathionylthiyl radicals, respectively.

For cystine, as well as for DMDS and GSSG, the later evolution of the spectra indicates secondary reaction steps which are in stark contrast to results from gas‐phase experiments.^[48]^ This observation clearly points to an involvement of the solvent environment. The spectral changes that appear within 3 ps rule out diffusive processes. Thiyl radicals will not react with the surrounding solvent molecules and we don't expect to produce sufficiently large amounts of solvated electrons at our experimental peak power densities to produce detectable concentrations of reduced species.^[57]^ Instead, we consider geminate recombination to be the cause for secondary reaction product(s). The similarity in spectral evolution between cystine and DMDS/GSSG leads us to associate this secondary reaction product with the perthiyl radicals of DMDS and GSSG, the methylperthiyl and glutathionylperthiyl radicals.

**Figure 3:**
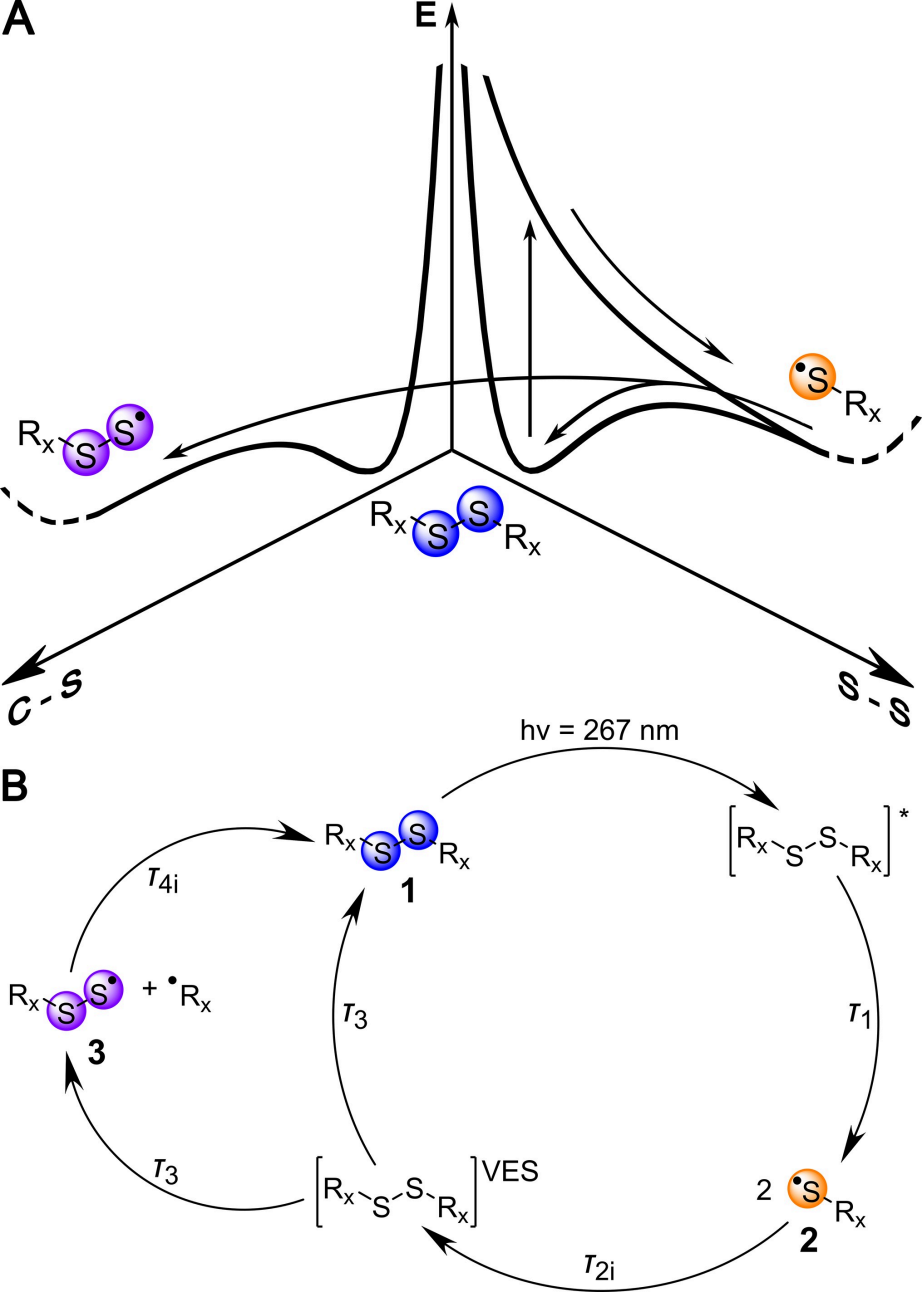
**A**) Illustration of the initial and the proposed secondary reaction pathway. The disulfide molecule (**1**) is split homolytically along the S−S bond direction upon UV excitation (vertical arrow), resulting in the formation of two identical thiyl radicals (**2**). Geminate recombination of these thiyl radicals occurs at the bond energy of about 3 eV above the energy minimum of the parent disulfide (**1**). The S−S‐ bond dissociation energy is about 0.5 eV higher than the one of the C−S bond,[^52^, ^67^, ^68^, ^69^, ^70^, ^71^, ^72^, ^73^, ^74^] rendering production of a perthiyl radical (**3**) energetically possible. Along both bond directions the solvent cage (dashed lines) creates potential barriers for cage escape of the radicals. Alternatively, the reformed disulfide (**1**) relaxes by intramolecular vibrational energy redistribution (IVR). **B**) Scheme of the photochemical cycle for the three symmetric aliphatic disulfides with residues R_DMDS_, R_Cystine_, and R_GSSG_. The S−S bond in **1** breaks homolytically upon 267 nm excitation, yielding two thiyl radicals (**2**, orange). Upon geminate recombination the parent molecule is in a highly vibrationally excited state (VES) from which we suggest C−S bond cleavage to yield a perthiyl radical (**3**, purple). Alternatively, **1** is reformed by intramolecular vibrational energy redistribution. From **3** and the respective carbonyl radical **1** will be reformed as well.

Energetically, such a process would be possible because the S−S bond dissociation energy is significantly above the C−S bond dissociation energy, as is illustrated in Figure 3A.[^52^, ^67^, ^68^, ^69^, ^70^, ^71^, ^72^, ^73^, ^74^] Upon a dipole‐allowed transition to an antibonding π* orbital of the S−S bond (vertical arrow) and subsequent homolytic bond cleavage two thiyl radicals (**2**) emerge. The repelling forces between the geminate products will be partially counteracted by interaction of these photoproducts with the surrounding solvation shell. The latter forms a complex and fluctuating energy landscape with barriers that facilitate product recombination at about 3 eV above the minimum energy of the parent (**1**), enough to render C−S bond cleavage and thereby formation of perthiyl radicals (**3**) possible. Thiyl radical acceleration will be inversely proportional to its mass, and we expect larger product separation for the lighter thiyl radicals (and a higher yield of solvent cage escape). Conversely, we expect to measure higher rates and yields of geminate recombination for heavier thiyl radicals.

In summary of the spectral data in Figure 2, the first step in the photochemistry of aliphatic disulfides upon 267 nm excitation is exclusive homolytic S−S bond cleavage. The formed thiyl radicals recombine to reform the parent molecule which will initially be in a highly vibrationally excited state that is almost 3 eV above the ground‐state energy minimum. From this state, relaxation to the disulfide ground state by intramolecular vibrational energy redistribution is possible. An alternative pathway to perthiyl formation exists that could explain formation of the secondary reaction products.

### Temporal Product Evolution

**Figure 4:**
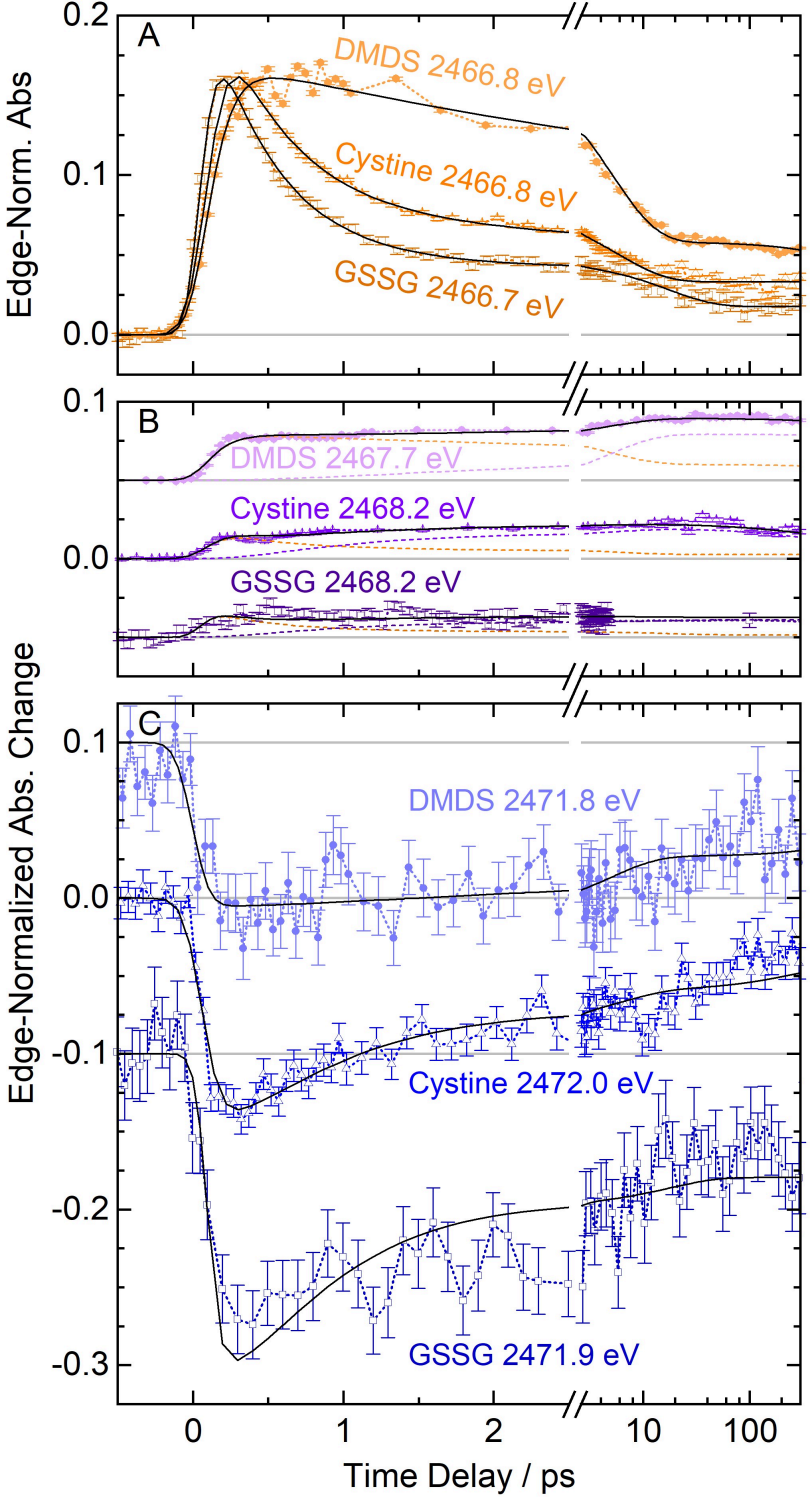
Delay scans of the relative absorption change up to 300 ps at the three indicated spectral positions. The delay scans of cystine are taken from a previous publication.^[56]^ After the break at 3 ps the delay axis changes from linear to logarithmic scale with minor tick marks starting at 4 ps. Fits of a rate‐equation model are plotted in black. **A**) Induced absorption at 2466.8 eV upon excitation of DMDS (light orange circles), cystine (orange triangles) and GSSG (dark orange squares). **B**) Induced absorption at 2467.7 eV and 2468.2 eV, respectively upon excitation of DMDS (light purple circles), cystine (purple triangles) and GSSG (dark purple squares). At 2468.2 eV, the thiyl radical contributes with 8 % of its maximum value to the signal from cystine (orange dashed line) and GSSG (dark orange dashed line) excitation. For DMDS measurements (light orange dashed line) at 2467.7 eV the methylthiyl radical absorption adds 17 % of its peak value. Hence, these scans are modeled as a sum of thiyl and secondary product contributions (from light to dark purple dashed line: DMDS, cystine and GSSG). **C**) Bleach signals at 2471.8 eV indicate a transient loss of the parent molecules DMDS (light blue circles), cystine (blue triangles) and GSSG (dark blue squares).

The spectral behavior in Figure 2 is reflected in the delay scans of the absorption changes that are plotted in Figure 4 for the three indicated probe energies which were chosen because they are indicative of the primary and secondary photoproducts as well as the loss of the parent molecules. By modeling these delay scans, we quantify chemical reaction rates and relative quantum yields.

Figure 4A shows the delay traces taken at 2466.8 eV which follow the evolution of the signals that we identify with the absorption of thiyl radicals produced by symmetrically splitting the disulfides. Here, the differences in the temporal evolution of the photochemistry of the three disulfides are most apparent: The population of the smallest thiyl species, the methylthiyl radical, grows in slower than the cysteinylthiyl and glutathionylthiyl radicals, and it persists considerably longer than the populations of the other two thiyl species. The methylthiyl radical population decays to 50 % of the peak signal within about 8 ps, whereas the cysteinylthiyl radical relaxes to 50 % within 2 ps. The glutathionylthiyl radical reaches 50 % signal loss within the first picosecond. Further signal decay occurs on picosecond timescales for the cysteinylthiyl and glutathionylthiyl radicals. At the longest time delay of 300 ps, the methylthiyl radical signal has decayed to a third of the peak signal while the cysteinylthiyl and glutathionylthiyl radical signals are about half that value.

Figure 4B shows the evolution of the delayed induced absorption shoulder at 2467.7 eV (DMDS) and 2468.2 eV (cystine, GSSG), which we attribute to the emergence of perthiyl radicals. This spectral position is considerably overlapping with the Lorentzian tail of the absorption peak at 2466.8 eV. This overlap masks the delayed growth of the secondary photoproduct absorption that is clearly seen in the differential spectra in Figure 2. From our lineshape analysis of the Lorentzian absorption peaks at 300 fs delay, we calculate a contribution of 8 % of the thiyl radical population at 2468.2 eV where we probe the secondary product. At the probe energy of 2467.7 eV, the methylthiyl radical contributes 17 % of its population to the observed signal. The decay of this secondary product signal has previously been studied in DMDS^[42]^ and cystine^[57]^ by some of the authors. For GSSG, we recorded a pump‐probe spectrum at 1 ns delay and within experimental error we could not observe a signal decay at 2468.6 eV compared to the signal at 100 ps.

Figure 4C displays the temporary loss of absorption caused by a loss of the parent disulfide molecules. After the bleaching has fully developed within the experiment's time resolution, the bleach signals decay on multiple timescales. While cystine and GSSG bleach signals decay to two thirds of their largest negative value within 2 ps, and a third of the bleach maxima remain at 300 ps, the DMDS bleach signal does not change appreciably within the first 3 ps and decays much slower than the bleach signals of the larger disulfides. Clearly, DMDS reformation is considerably slower than reformation of cystine and GSSG. We note that the relatively large error of the bleach signals compared to induced absorption signals at lower probe energy is due to quantification of absorption by total fluorescence yield, where the largest absorption of the parent molecules exists and signal fluctuations are strongest (see section 7 of SI for details).

To model the temporal evolution of the photoproducts, we employ a combination of a four‐state and a five‐state kinetic model that was previously used for the analysis of the cystine kinetics.^[57]^ This model is based on the observation that most of the thiyl radical signal and parent molecule bleaching relax on two observable timescales but not in the same temporal fashion. And the induced absorption that signals a secondary product, rises on the same two timescales. Therefore, the thiyl radicals – created from the initially excited state – cannot exclusively decay into the parent compound but their population decay must also lead to the secondary product. The vibrationally excited state of the parent compound, created by product recombination, is a short‐lived intermediate state in the model. From this state, the secondary product emerges with a certain yield that competes with thermalization of the parent compound.

The model represents a photochemical cycle as depicted in Figure 3B with exponential time constants $\tau_{1}$ for thiyl radical formation, $\tau_{2i}$ for thiyl radical lifetimes, $\tau_{3}$ for the VES intermediate lifetime, and $\tau_{4i}$ for the secondary product lifetimes. Fitting this model to the three sets of disulfide delay scans results in the black model traces in Figure 4. Some populations persist much longer than the longest delay of 300 ps in Figure 4 which is why we have fixed some of the time constants to a value much larger than 300 ps. Specifically, we have also fixed the time constants for secondary product decay, $\tau_{41,DMDS}$ = 4.5 ns^[42]^ and $\tau_{41,GSSG}\approx \infty$ , while for cystine we have fit $\tau_{41,Cystine}$ to the published data which extends to 800 ps and set $\tau_{41,Cystine}\approx \infty$ . All optimized time constants and relative yields are listed in Table 1.

**Table 1:** Optimized global fit parameters of the kinetic model for the delay scans of absorption changes in DMDS, cystine and GSSG.

Process	Time constant	DMDS	Cystine	GSSG	Relative yield	DMDS	Cystine	GSSG
thiyl formation	$\tau_{1}/\mathrm{ps}$	0.12±0.01	0.06±0.02	0.05±0.02	$\varphi_{1}$	1	1	1
thiyl decay	$\tau_{21}/\mathrm{ps}$	–	0.49±0.03	0.45±0.02	$\varphi_{21}$	–	0.65±0.01	0.79±0.01
	$\tau_{22}/\mathrm{ps}$	5.0±0.17	6.1±0.5	17±3	$\varphi_{22}$	0.66±0.01	0.20±0.01	0.13±0.01
	$\tau_{23}/\mathrm{ps}$	3300±290	$>10^{4}\;\left(\mathrm{fixed}\right)$ *	$>10^{4}\;\left(\mathrm{fixed}\right)$ *	$\varphi_{23}$	$1-\varphi_{22}$	$1-\varphi_{21}-\varphi_{22}$	$1-\varphi_{21}-\varphi_{22}$
parent reformation	$\tau_{3}/\mathrm{ps}$	0.47±0.12	0.43±0.05	0.43±0.11 **	$\varphi_{31}$	0.46±0.21	0.68±0.01	0.66±0.03
perthiyl formation					$\varphi_{32}$	$1-\varphi_{31}$	$1-\varphi_{31}$	$1-\varphi_{31}$
perthiyl decay	$\tau_{41}/\mathrm{ps}$	$4500\;\left(\mathrm{fixed}\right)$ ^[39]^	250±120	$>10^{4}\;\left(\mathrm{fixed}\right)$ *	$\varphi_{41}$	$1\;\left(\mathrm{fixed}\right)$	0.42±0.09	$1\;\left(\mathrm{fixed}\right)$
	$\tau_{42}/\mathrm{ps}$	–	$>10^{4}\;\left(\mathrm{fixed}\right)$ *	–	$\varphi_{42}$	–	$1-\varphi_{41}$	–

#### Thiyl Radical Formation

For DMDS we find a thiyl radical formation time constant of $\tau_{1,DMDS}$ =120 fs which is the value reported by Schnorr et al. for UV‐excited gas‐phase DMDS.^[48]^ The thiyl radical signal for cystine and GSSG appears to rise significantly faster with $\tau_{1,Cystine}$ =63 fs and $\tau_{1,GSSG}$ =54 fs (we note that $\tau_{1,Cystine}$ is smaller than we previously reported due to an adjusted data selection for the fitting procedure that results in a slightly better agreement with experimental data, c.f. SI). All reported errors are derived from the Hessians of the fitting procedure. The model also contains widths of the instrument response function. These are similar to the thiyl formation times $\tau_{1}$ . We therefore consider the reported errors as lower bounds, and the values for $\tau_{1}$ not to be in contradiction to those previously reported. The significantly slower formation time of the methylthiyl radical suggests that the potential energy surfaces along the reaction coordinate are not identical among the three disulfides. Instead, the two larger disulfides appear to have steeper gradients along the S−S bond onto which the ground‐state wavepacket is projected by the UV excitation. However, DMDS is measured in methylcyclohexane which has an X‐ray attenuation length of 90 μm at 2.5 keV, three‐fold larger than water. Group velocity mismatch between pump and probe pulses may cause additional broadening in the DMDS measurements. We also note that the wavepacket propagation, that leads to thiyl radical formation, is ballistic, but we model this step kinetically. The wavepacket dispersion will increase the timing uncertainty in radical formation which we describe by an exponential radical formation time.

#### Ultrafast Radical Decay

Thiyl radical recombination is the dominant sub‐picosecond process for cystine and GSSG with similar time constants of $\tau_{21,Cystine}$ =0.49 ps and $\tau_{21,GSSG}$ =0.45 ps. The ultrafast recombination times for these larger disulfides suggest that solvent interactions largely manifest in confinement. The sulfur atoms remain in close proximity and their charge density does not relax much, i. e. the S−S bond can reform rapidly. The high quantum yields of $\varphi_{21,Cystine}$ =0.65 and $\varphi_{21,GSSG}$ =0.79 underscore the effectiveness of ultrafast geminate radical recombination in aliphatic disulfides as small as cystine. Aromatic disulfides of similar weight do not exhibit such ultrafast S−S bond reformation due to rehybridization of the sulfur charge density in the products.^[75]^

#### Picosecond Kinetics

In contrast, methylthiyl radical decay is an order of magnitude slower and well described by an exponential time constant of $\tau_{22,DMDS}$ =5 ps with a quantum yield of $\varphi_{22,DMDS}$ =0.66. This observation suggests that the mutually repulsive methylthiyl radical acceleration is large enough to reach a critical separation such that solvent molecules form a potential energy barrier that prohibits ultrafast recombination. We consider solvent involvement such that the thiyl radicals have either rotated (and need to rotate back for disulfide reformation) and/or solvent molecules from the first solvation shell are partially intercalated between the sulfur atoms. The picosecond relaxation also exists for the cysteinylthiyl and glutathionylthiyl radicals and it exhibits a size dependence that is opposite to the ultrafast (sub‐picosecond) thiyl radical decay, with time constants of $\tau_{22,Cystine}$ =6.1 ps and $\tau_{22,GSSG}$ =17 ps at minority quantum yields of $\varphi_{22,Cystine}$ =0.20 and $\varphi_{22,GSSG}$ =0.13. While these picosecond time constants suggest that a sub‐ensemble of thiyl radicals with larger separation exists that involves additional interactions with solvent molecules, we have to consider orientational relaxation which can influence our pump‐probe signals because the polarizations of pump and probe pulses in our experiment were parallel to each other. The UV pulse preferentially excites molecules with S−S bonds parallel to the UV polarization, and the angle between the S−S bond direction and the transition dipoles moment for the lowest X‐ray transition of the parent, the thiyl and the perthiyl radical is within 20° (c.f. SI). Therefore, molecular rotation will lead to a signal decrease. Time‐resolved fluorescence anisotropy studies have reported orientational relaxation times of tens of picoseconds for fluorescent (aromatic) molecules of mass similar to cysteinylthiyl radicals.^[76]^ These numbers preclude a significant pump‐probe signal decay due to orientational relaxation. However, femtosecond absorption spectroscopy studies have found shorter orientational decay times, suggesting that the picosecond decay constants may indeed be influenced by orientational relaxation.^[77]^ An indicator of population decay rather than orientational relaxation is the monotonically increasing signal of the secondary product that we attribute to perthiyl radical population. Orientation relaxation would decrease the perthiyl absorption signal because the dipole moments of the lowest X‐ray transitions are almost parallel to the S−S bond. We conclude from this discussion that the picosecond time constants $\tau_{22}$ primarily describe thiyl radical recombination that involves interfering solvent molecules and a barrier along one or several reaction coordinates (translational and rotational) that needs to be overcome by thiyl radicals in close proximity of each other. The size‐dependence is such that the lighter methylthiyl radicals recombine faster than the larger thiyl radicals ($\tau_{22,DMDS}<\tau_{22,Cystine}<\tau_{22,GSSG}$ ).

#### Parent Reformation vs. Secondary Product Formation

Within our model, DMDS reformation occurs with $\varphi_{31}$ =46 % probability while parent reformation probability increases to about two thirds for the larger thiyl radicals. This size‐dependence may be explained by the smaller number of vibrational modes in DMDS compared to cystine and GSSG. The excess energy in the reformed parent molecules will be dissipated into these modes during the ensuing thermalization process. Cystine and GSSG show little difference in their reformation yields $\varphi_{31}$ . This modeling result can be rationalized by GSSG being a tripeptide dimer with the cystine moiety at its center. The number of modes that are strongly coupled to S−S bond modes should not be appreciably larger in GSSG than in cystine. The competing process of secondary product formation leads to longer‐lived species with (sub‐)nanosecond time constants. The preceding processes smear out the moment of secondary product formation which means that if a secondary product pair is generated, e. g. a perthiyl and a carbonyl radical, fast recombination is unlikely to be observable. We only observe the portion that becomes sufficiently separated to require diffusion in order to reform the parent compound which may explain the relatively slow decay of the secondary products.

#### Final Considerations

In our previous publication, we argued that the high yield of (ultrafast) S−S bond reformation in cystine demonstrates the ability of aliphatic disulfides to dissipate the high‐energy quantum of UV photons very effectively by bond cleavage and geminate recombination.^[57]^ Here we find this effect to be even stronger for glutathione disulfide. This finding further points towards a dynamical photostability of the aliphatic disulfide bond motif in large molecular systems such as disulfide‐stabilized proteins. This property may also be found in or transferred to materials containing disulfide bonds. The lower recombination rates and yields of DMDS demonstrate that smaller products can evade ultrafast recombination. According to our model, DMDS is more likely to form secondary products than to thermally equilibrate as DMDS within the first 300 ps. This behavior we attribute to the comparatively small number of vibrational modes into which energy can be dissipated upon recombination. Concerning the role of DMDS in atmospheric photochemistry, S−S bond dissociation in solution will probably not be of high relevance because DMDS as a photoactive sulfur species generally undergoes chemical transformations in the gas phase and reactions in tropospheric water droplets are largely excluded due to the poor water solubility of DMDS and dimethyl sulfide (DMS), the latter being by far the most abundant sulfur compound in the earth's atmosphere.^[12]^

## Conclusions

We have investigated the UV photochemistry of aliphatic disulfides in solution by comparing dimethyl disulfide and glutathione disulfide to cystine. The response to 267 nm excitation is probed by femtosecond X‐ray absorption spectroscopy at the sulfur K‐edge. In these three aliphatic disulfides UV absorption leads exclusively to homolytic S−S bond cleavage. Solvent‐mediated geminate recombination occurs at majority quantum yield within the observed time‐frame of 300 ps, leading to the reformation of the parent disulfides. The larger the residue, the more effective geminate recombination is. We find three recombination regimes (ultrafast, picosecond, nanosecond) that we attribute to the potential energy landscape created by the solvent environment: in the first regime, the solvent cage provides a first barrier which effectively increases with residue size and which leads to strong confinement and ultrafast recombination. In the second regime, the first barrier is overcome but the geminate radical pair is still confined by the solvent environment (constituting a second barrier with a more generalized reaction coordinate which could also be rotational in nature) such that their close proximity permits disulfide recombination on picosecond timescales. The third regime we ascribe to fully separated products which recombine by diffusive motion. Dimethyl disulfide does not exhibit ultrafast (<1 ps) recombination times which we attribute to a larger mutual acceleration of the thiyl fragments (due to their smaller mass), thereby always overcoming a first solvation shell barrier and precluding ultrafast recombination.

In the three studied disulfides we observe the delayed formation of a secondary product that we assign to the respective perthiyl radicals. Energetically, these can form during thiyl radical recombination because the dissociation energy of the C−S bonds is lower than the dissociation energy of the S−S bond. The spectroscopic evidence is not as unambiguous as for the thiyl radicals but as discussed in Ochmann et al.,^[57]^ other chemical species cannot form for energetic reasons and/or do not exhibit absorption in the observed spectroscopic range.

The very effective and inherent radical quenching ability of condensed phase thiyl radicals via radical recombination points towards a possible activity of protein sulfur groups in radical quenching reactions. To fully understand their photochemical functionality, it will be necessary to directly investigate disulfide bonds within proteins where there is no immediate solvent cage but instead, backbone motion which influences the recombination probability of thiyl radicals.^[78]^ Furthermore, in proteins the common proximity of disulfide bonds and aromatic residues can open additional reaction pathways such as energy or charge transfer reactions that merit further investigation.

## Supporting Information

Experimental data processing is detailed in the Supporting Information. The authors have cited additional references within the Supporting Information.[^57^, ^79^, ^80^, ^81^, ^82^, ^83^, ^84^, ^85^, ^86^]

## Conflict of Interests

The authors declare no conflict of interest.

## Supporting information

As a service to our authors and readers, this journal provides supporting information supplied by the authors. Such materials are peer reviewed and may be re‐organized for online delivery, but are not copy‐edited or typeset. Technical support issues arising from supporting information (other than missing files) should be addressed to the authors.

Supporting Information

## Data Availability

The data that support the findings of this study are available from the corresponding author upon reasonable request.
