# Supplementary material for: Size‐Dependent Ultrafast UV Photochemistry of Aliphatic Disulfides in Solution
Source: Chemistry. 2025 May 26;31(34):e202404695. doi: 10.1002/chem.202404695 (PMC12172612; doi:10.1002/chem.202404695)
Supplement: Supplementary file 1 — Supporting Information [file CHEM-31-e202404695-s001.pdf]

Chemistry–A European Journal

Supporting Information

Size-Dependent Ultrafast UV Photochemistry of Aliphatic Disulfides in Solution

Jessica Harich, Rory Ma, Miguel Ochmann, Antonia Freibert,* Yujin Kim, Minseok Kim, Eunhyo Kim, Madhusudana Gopannagari, Junho Lee, Tae Gyun Woo, Haneol Oh, Ru-Pan Wang, Jae Hyuk Lee, Tae Kyu Kim,* and Nils Huse*

Size-Dependent Ultrafast UV Photochemistry of Aliphatic Disulfides in Solution

Jessica Harich,[a],* Rory Ma,[b],* Miguel Ochmann,[a],* Antonia Freibert,*[a] Yujin Kim,[b] Minseok Kim,[b] Eunhyo Kim,[c] Madhusudana Gopannagari,[c] Junho Lee,[c] Tae Gyun Woo,[c] Haneol Oh,[c] Ru-Pan Wang,[a] Jae Hyuk Lee,[b] Tae Kyu Kim*[c] and Nils Huse*[a]

[a] J. Harich, Dr. Miguel Ochmann, Dr. Antonia Freibert, Dr. Nils Huse
Department of Physics
University of Hamburg
Center for Free-Electron Laser Science
Luruper Chaussee 149, 22761 Hamburg, Germany
E-mail: nils.huse@uni-hamburg.de, antonia.freibert@physik.uni-hamburg.de

[b] Dr. R. Ma, Dr. Y. Kim, Dr. M. Kim, Dr. J.H. Lee
Pohang Accelerator Laboratory
POSTECH
Pohang 37673, Republic of Korea

[c] E. Kim, M. Gopannagri, J. Lee, T.G. Woo, H. Oh, Dr. T.K. Kim
Department of Chemistry
Korea Advanced Institute of Science and Technology (KAIST)
Daejeon 34141, Republic of Korea
E-mail: taekyu.kim@kaist.ac.kr

Supporting Information

1. **Theory calculations of DMDS and GSSG**
   - Fig. S1 Theory calculations and lineshape analysis of DMDS and GSSG.
2. **Lineshape analysis of DMDS and GSSG reaction products**
   - Tab. S1 Voigt parameters of the lineshape of the glutathionylthiyl radical.
   - Tab. S2 Voigt parameters of the lineshape of the methylthiyl radical.
   - Tab. S3 Voigt parameters of the static absorption spectrum of GSSG.
   - Tab. S4 Voigt parameters of the static absorption spectrum of DMDS.
3. **Delay scan composition**
   - Fig. S2. Composition of GSSG and DMDS delay scans.
   - Fig. S3. Zoom in on the DMDS delay scan and fit curve.
4. **Kinetic model**
   - Fig. S4. Schematic of the generalized kinetic fitting model for all three disulfide species.
   - Tab. S5: Optimized parameters of the rate-equation model for DMDS.
   - Tab. S6: Optimized parameters of the rate-equation model for GSSG.
5. **Point omission in cystine data**
   - Tab. S7: Optimized parameters of the rate-equation model for Cystine.
   - Tab. S8: Optimized parameters of the rate-equation model for Cystine without masked points.
   - Fig. S5. Comparison of Cystine fit curves obtained by global fitting to all three Cystine delay.
6. **Orientation of transition dipole moments**
   - Tab. S9: Angles between transition dipoles and bond directions in L-cystine.
7. **Filtering of measured intensity data**
   - Fig. S6. Correlation plot of measured incident to X-ray fluorescence intensity

1. Theory Calculations of DMDS and GSSG

Equilibrium structure optimizations for all molecular species related to DMDS and Cystine were carried out using the PBE functional[79,80] in combination with the def2-TZVP basis set,[81] as implemented in the quantum chemistry software package Gaussian.[82] For species related to GSSG, the Conformer-Rotamer Ensemble Sampling Tool (CREST) within the xtb program was employed.[83] followed by a quantum chemical re-optimization at the PBE/def2-TZVP level of theory. The conformer with the lowest energy was selected for further analysis. The atomic coordinates for all optimized structures are available in the Supplementary Data.

The X-ray absorption spectra for all molecular species were simulated using time-dependent density functional theory (TD-DFT) at the PBE0/def2-TZVP(-f) level,[84] incorporating the RIJCOSX approximation, using the quantum chemistry program Orca[85,86]. The conductor-like polarizable continuum model (CPCM) with water as the solvent for cystine and GSSG and cyclohexane as the solvent for DMDS was applied. To align the calculated transition energies with experimental data, the computed transition energies were shifted by 52.05 eV (DMDS), 52.09 eV (cystine) and 51.77 eV (GSSG), respectively.

The calculated transitions for DMDS and GSSG are shown in Fig. S1. In panel A, the calculated lowest vertical excitation energies of DMDS (grey sticks) and GSSG (dark green sticks) match the experimental spectra well. The convolution of these δ-distributions with Voigt profiles is shown in the same panel as blue lines (c.f. section 2). In panel B, the lowest induced absorption at 2466.8 eV observed 300 fs after excitation of DMDS and GSSG can be identified as the respective thiyl radicals (orange sticks). The shoulder to the energetically higher side of the primary induced absorption, observed 3 ps after excitation, matches the calculated transitions for perthiyl radicals (purple sticks).

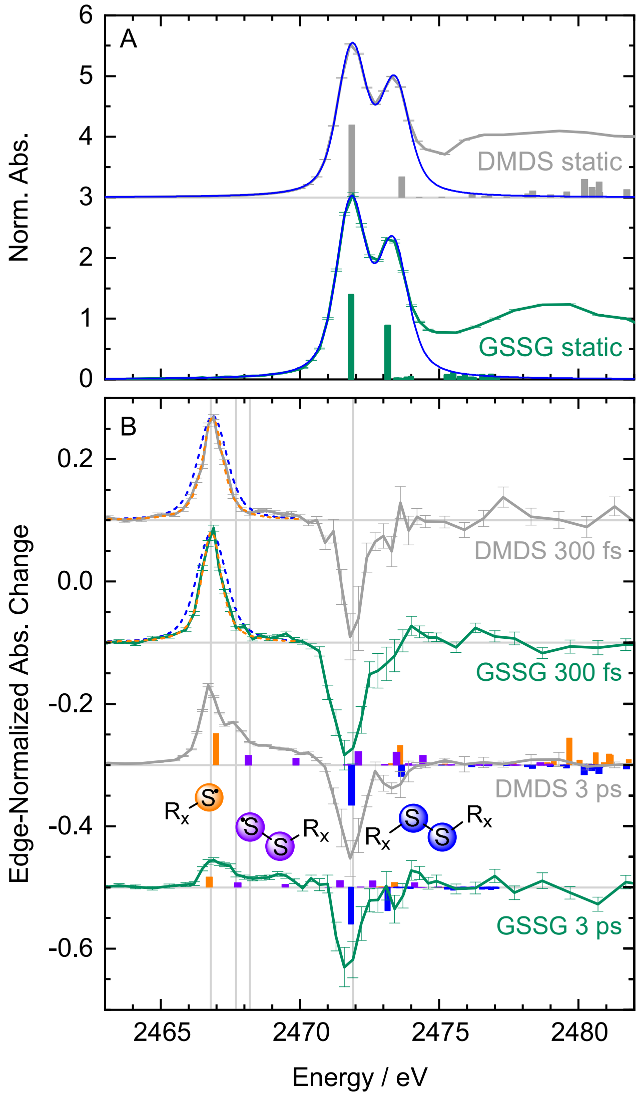

Figure S1. Static and differential sulfur K-edge absorption spectra of Dimethyl disulfide (DMDS, grey) and Glutathione disulfide (GSSG, dark green). **A.** Static spectra of DMDS and GSSG with fits of a sum of two Voigt profiles to the experimental data between 2463 eV and 2475 eV. In addition, calculated vertical excitation energies are shown as sticks (DMDS: grey sticks, GSSG: dark green). **B.** Differential spectra at 300 fs and 3 ps temporal delay after 267 nm excitation of DMDS (300 fs offset by +0.1, 3 ps offset by -0.3) and GSSG (300 fs offset by -0.1, 3 ps offset by -0.5). Vertical grey lines indicate the energies at which delay scans were recorded. Voigt profiles (dashed orange curves, fixed Lorentzian-FWHM of 0.66 eV) were fit to the measured absorption lineshapes at 2466.8 eV and 300 fs delay. For comparison, Voigt profiles (dashed blue curves) with lineshape parameters obtained from fits of Voigt profiles to the static spectra in panel A are shown, too. The calculated transitions for reaction products of DMDS and GSSG present after 3 ps are shown as colored sticks: parent bleach signals (blue) and the respective thiyl (orange) and perthiyl (purple) radicals.

2. Lineshape analysis of DMDS and GSSG reaction products

For the three investigated disulfides the energetically lowest induced absorption feature can be seen in Fig. S1B at 2466.8 eV at 300 fs after excitation. This absorption feature has a Lorentzian lineshape that extends to about 2469 eV. The respective signal strengths of this lineshape in percent of its peak values at the energies of the secondary induced absorption features amount to 8.18 % at 2468.2 eV for Cystine,[57] 8.13 % at 2468.2 eV for GSSG and 17.3 % at 2467.7 eV for DMDS.

We previously extracted the Lorentzian lifetime broadening of the lowest cysteinylthiyl lineshape with $\mathrm{FWHM_L}$ = 0.66 eV. We can extract the Si(111) double-crystal monochromator (DCM) bandwidth $\mathrm{FWHM_G}$ fitting a Voigt profile to the lowest induced absorption features of UV-excited DMDS and GSSG with $\mathrm{FWHM_L}$ = 0.66 eV as a fixed lineshape parameter (orange dashed curves in Fig. S1B). We find Gaussian widths of the glutathionylthiyl and methylthiyl radical peaks of $\mathrm{FWHM_{G,GSSG}} = 0.37\ \mathrm{eV}$ and $\mathrm{FWHM_{G,DMDS}} = 0.42\ \mathrm{eV}$, respectively. Both values are close to optimal Si(111) DCM bandwidth of 0.35 eV. The Voigt lineshape parameters obtained for DMDS and GSSG thiyl radicals are listed in Tables S1 and S2.

Table S1: Voigt parameters of the lineshape of the glutathionylthiyl radical at 2466.8 eV with a fixed Lorentzian width of $\mathrm{FWHM_L} = 0.66\ \mathrm{eV}$, yielding a Gaussian width of $\mathrm{FWHM_{G,GSSG}} = 0.37\ \mathrm{eV}$.

Model	Voigt
Equation	y = nfl_voigt(x,y0,xc,A,wG,wL)
y0	0 ± 0
xc / keV	2.46682 ± 1.23534E-5
$\mathrm{FWHM_{G,GSSG}}$ / keV	3.6915E-4 ±5.90594E-5
FWHM$_L$ / keV	6.6E-4
A	2.2052E-4± 5.27971 E-6
Reduced Chi-Sqr	3.11596930252
R-Square (COD)	0.99054726223726

Table S2: Voigt parameters of the lineshape of the methylthiyl radical at 2466.8 eV with a fixed Lorentzian width of $\mathrm{FWHM_L} = 0.66\ \mathrm{eV}$, yielding a Gaussian width of $\mathrm{FWHM_{G,DMDS}} = 0.42\ \mathrm{eV}$.

Model	Voigt
Equation	y = nfl_voigt(x,y0,xc,A,wG,wL)
y0	0 ± 0
xc / keV	2.46686 ± 1.48008E-5
$\mathrm{FWHM_{G,DMDS}}$ / keV	4.22145E-4 ± 6.01188E-5
FWHM$_L$ / keV	6.6E-4
A	2.11228E-4± 5.86164E-6
Reduced Chi-Sqr	3.85379724282
R-Square (COD)	0.98920693463446

We previously found the absorption spectrum of Cystine to have a distinctly broadened shape compared to the shape of the energetically lowest induced absorption at 2466.8 eV.[57] Fitting Voigt profiles to the first two transitions of the static spectra of DMDS and GSSG in Fig. S1A with a fixed natural linewidth of $\mathrm{FWHM_L} = 0.66\ \mathrm{eV}$ yields Gaussian widths of $\mathrm{FWHM_{G,DMDS}^{sum}} = 0.84\ \mathrm{eV}$ and $\mathrm{FWHM_{G,GSSG}^{sum}} = 0.87\ \mathrm{eV}$ respectively. The optimized parameters are listed in Tables S3 and S4, the fit curves are displayed in Fig. S1A (blue curves). To visualize the broadening of the lineshapes of the static spectra compared to the lineshapes of the primary induced absorption 300 fs after excitation, Voigt profiles using of the broadened Gaussians are displayed with the induced absorptions in Fig. S1B as blue dashed curves. Both widths are comparable to the Gaussian width previously reported for Cystine ($\mathrm{FWHM_{G,Cystine}^{sum}} = 0.86\ \mathrm{eV}$).[57] The Gaussian broadening due to spectral inhomogeneity of the sample absorption can now be calculated by

$$\mathrm{FWHM_G^{sample}} = \sqrt{(\mathrm{FWHM_G^{sum}})^2 - \left(\mathrm{FWHM_G^{Si(111)}}\right)^2} \quad (1)$$

giving $\mathrm{FWHM_{G,DMDS}^{sample}} = 0.76$ eV and $\mathrm{FWHM_{G,GSSG}^{sample}} = 0.80\ \mathrm{eV}$ for heterogeneous broadening. The value for GSSG and DMDS are close to that of L-Cystine which exhibits heterogeneous broadening of $\mathrm{FWHM_{G,cystine}^{sample}} = 0.79$ eV. We attribute this broadening to a distribution of dihedral angles which lead o slight shifts of the lowest sulfur-1s transitions.

Table S3: Voigt parameters for the first two transitions (Peak1 and Peak2) of the static absorption spectrum of GSSG with a fixed Lorentzian width of $\mathrm{FWHM_L} = 0.66\ \mathrm{eV}$, yielding a Gaussian width of $\mathrm{FWHM_{G,GSSG}^{sum}} = 0.87\ \mathrm{eV}$.

Model	Voigt	
Equation	y = nlf_voigt(x,y0,xc,A,wG,wL);	
Plot	Peak1	Peak2
y0	0 ± 0	
xc / keV	2.47183 ± 7.1917E-6	2.47334 ± 9.96699E-6
A	0.00473 ± 4.09457E-5	0.00352 ± 4.02499E-5
$\mathrm{FWHM_{G,GSSG}^{sum}}$ / keV	8.68165E-4 ± 1.55415E-6	
FWHM$_L$ / keV	6.6E-4	
Reduced Chi-Sqr	0.00141576309661	
R-Square (COD)	0.99901010174286	

Table S4: Voigt parameters for the first two transitions (Peak1 and Peak2) of the static absorption spectrum of DMDS with a fixed Lorentzian width of $\mathrm{FWHM_L} = 0.66\ \mathrm{eV}$, yielding a Gaussian width of $\mathrm{FWHM_{G,DMDS}^{sum}} = 0.84\ \mathrm{eV}$.

Model	Voigt	
Equation	y = nlf_voigt(x,y0,xc,A,wG,wL);	
Plot	Peak1	Peak2
y0	0 ± 0	
xc / keV	2.47186 ± 2.31539E-6	2.4734 ± 3.43777E-6
A	0.00394± 1.12813E-5	0.00297 ± 1.13354E-5
$\mathrm{FWHM_{G,DMDS}^{sum}}$ / keV	8.40165E-4 ± 5.18134E-6	
FWHM$_L$ / keV	6.6E-4	
Reduced Chi-Sqr	1.11407621291E-4	
R-Square (COD)	0.99989113268749	

3. Delay scan composition

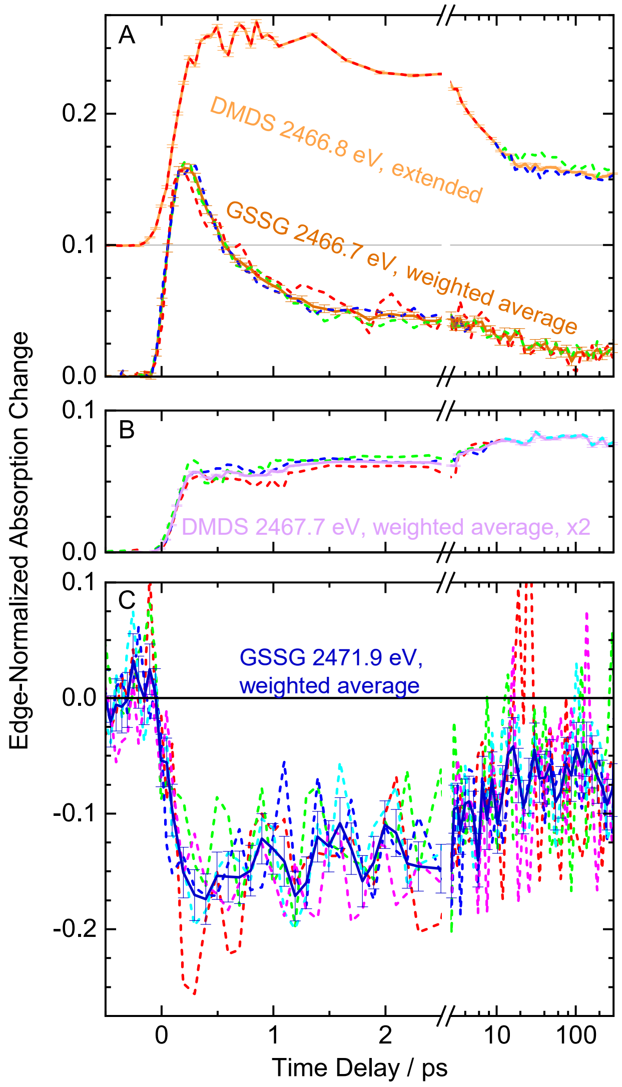

Figure S2. Composition of GSSG and DMDS delay scans. **A.** DMDS delay scan at 2466.8 eV (orange) composed of three separate measurements: one scan from -1ps to 10 ps (dashed red line) and two scans are from 10 ps up to 300 ps (dashed green and blue lines). The GSSG delay scan at 2466.7 eV (brown) is a weighted average of three scans (blue, green, red) from -1 ps to 5 ps and of two scans (green, red) from 5 ps to 300 ps. **B.** The DMDS delay scan at 2467.7 eV (light purple) is the weighted average of three scans from -1 to 10 ps (dashed blue, red, green). One scan extends to 300 ps (cyan). **C.** GSSG delay scan at 2471.9 eV (dark blue) as the weighted average of five delay scans from -1 ps to 300 ps (dashed blue, green, red, cyan, pink).

The delay scans of GSSG at the spectral positions 2466.7 eV and 2471.9 eV are the averaged of multiple scans (Fig. S2), weighted by the inverse variance of each measured data point: The delay scan of GSSG at 2466.7 eV (Fig. S1A, brown) is the average from three separate measurements (blue, green, red) in the range from -1 ps to 5 ps. In the range from 5 ps up to 300 ps the presented delay scan is averaged from two of those scans (green, red). The scan at 2471.9 eV (Fig. S1C, dark blue) is a weighted average from five delay scans over the full measurement range (blue, green, red, cyan, pink). Due to the non-linear scan file and slight shifts of time zero between measurements, the separate scans have been linearly interpolated onto a common time-axis. All scans were measured at a power density of 0.46 TW/cm^2.

The delay scans of DMDS at 2466.8 eV and 2467.7 eV are the averages of multiple scans. The thiyl scan at 2466.8 eV (Fig. S1A, orange) is a single scan (red) taken at a power density of 0.18 TW/cm^2 in the range from -1 ps to 10 ps which is extended by a weighted average of two scans (blue, green) from 10 ps to 300 ps, taken at 0.25 TW/cm^2 and linearly interpolated onto a common time axis. The delay scan at 2467.7 eV is a weighted average of four separate measurements (blue, green, red, cyan) in the range from -1 ps to 10 ps linearly interpolated onto a common time-axis. One delay scan extends to 300 ps (cyan).

Figure S3 shows the first picoseconds of the DMDS bleach delay scan. The representation in Fig.1 (starting from -0.5 ps) seems to indicate a y-offset but the measured delay range from -1 ps onwards shows that a noisy, yet correct, baseline is assigned.

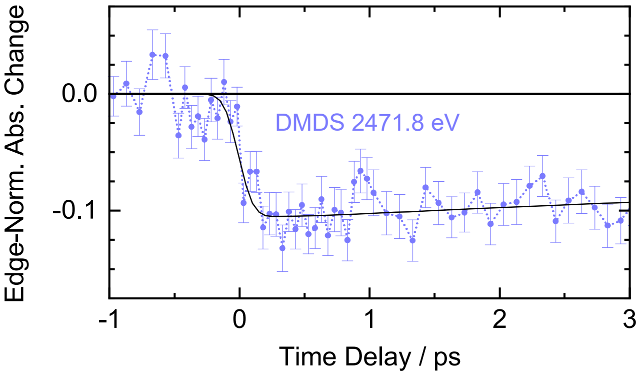

Figure S3. DMDS delay scan (dark blue) and fit curve (black) at 2471.9 eV as shown in Fig. 1 but from -1 ps to 0 ps to show the correct baseline assignment at negative delays.

4. Kinetic Model

The behavior of the experimental delay scans of DMDS, GSSG and Cystine have been modelled by a system of rate-equations as shown in Fig. S3, based on the kinetic model developed by Ochmann et al. of Cystine.[57] After UV-excitation the exited state (N_1) population decays when symmetrically breaking the disulfide bond results in the formation two identical thiyl radicals (N_2). A small amount of these thiyl radicals is stable on the observed time scales (rate constant $k_{23} = 0$). The remaining N_2 population decays biexponentially (rate constants k_{21} and $k_{22} > k_{21}$), re-populating the electronic ground state surface of Cystine at high vibrational energy. From this vibrationally excited state (VES, N_3) either the parent molecule (N_0) is recovered cleavage of the C-S bond yields perthiyl and carbonyl radicals (N_4) with a rate constant k_3). The perthiyl / carbonyl radical pairs are either stable on the observed time scales (rate constant $k_{42} = 0$) or undergo recombination to reform the parent disulfide (rate constant k_{41}).

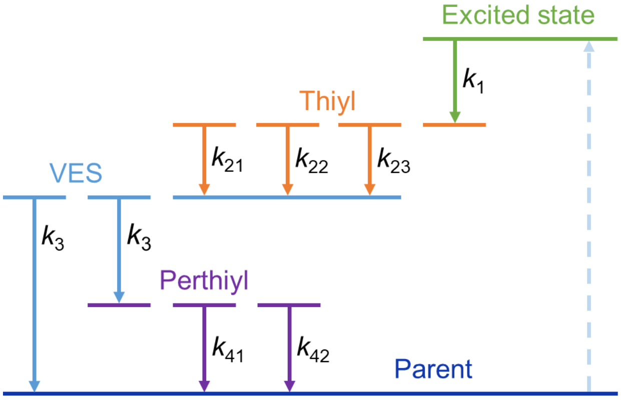

Figure S4. Schematic of the kinetic model for the three investigated disulfides, based on the model developed previously by Ochmann et al.[57] After 267-nm excitation, the excited state population (N_1, green) evolves with the rate constant k_1 into the first product state with a population N_2 (Thiyl, orange). The N_2 population decays as a statistical mixture of three subensembles with rate constants k_{21}, k_{22} and k_{23} into a short-lived, vibrationally excited state of the parent molecule with a population N_3 (VES, light blue). The population N_3 decays with the rate constant k_3 into either the ground state N_0 (Parent, blue) or a new product with population N_4 (Perthiyl, purple). N_4 decays as a statistical mixture of two subensembles with rate constants k_{41} and k_{42} - reforming the parent molecule.

The populations according to the above rate-model are provided in the following. Note that we introduce the simplification of a single rate constant k_3 regardless of where the VES population decays to. We define $k_1 \coloneqq t_1^{-1}$ and $k_{\mathrm{ij}} \coloneqq t_{\mathrm{ij}}^{-1}$ to link time constants to rate constants. All exponential decay terms are convolved with a Gaussian instrument response function, yielding Gaussian error functions:

$$e_{\mathrm{ij}} \coloneqq \exp\left(-k_{\mathrm{ij}}(t-t_0) + \frac{\sigma^2 k_{\mathrm{ij}}^2}{2}\right) \cdot \mathrm{err}\left(k_{\mathrm{ij}}\right), \tag{2}$$

with the error function defined as

$$\mathrm{err}\left(k_{\mathrm{ij}}\right) \coloneqq \frac{1}{2}\left(1 + \mathrm{erf}\left[\frac{1}{\sqrt{2}}\left(\frac{t-t_0}{\sigma} - \sigma k_{\mathrm{ij}}\right)\right]\right) \tag{3}$$

In the particular case of $k_{ij} \to 0$ we define

$$\mathrm{Err} \coloneqq \mathrm{err}(0) = \frac{1}{2}\left(1 + \mathrm{erf}\left[\frac{1}{\sqrt{2}}\left(\frac{t-t_0}{\sigma}\right)\right]\right) \tag{4}$$

We then obtain the following solutions for the populations.

$N_1(t)$:

The population $N_1(t)$ of the initially excited state is given by $N_1(t) = e_1$ with e_1 defined analogously to eq. 2 with k_1 replacing k_{ij}.

$N_2(t)$:

The population $N_2(t)$ of the thiyl radical, which grows at a rate k_1 consists of three sub-ensembles N_{21}, N_{22}, N_{23} that decay with the respective rates k_{21}, k_{22} and k_{23}. The yields of these populations are ϕ_{21}, ϕ_{22} and ϕ_{23} with $\phi_{23} = 1 - \phi_{21} - \phi_{22}$:

$$N_2(t) = \textstyle\sum_{i=1}^{3} \phi_{2\mathrm{i}}\, N_{2\mathrm{i}}(t) \quad \text{where} \quad N_{2\mathrm{i}}(t) = k_1 \left[\frac{e_1}{(k_{2\mathrm{i}}-k_1)} + \frac{e_{2\mathrm{i}}}{(k_1-k_{2\mathrm{i}})}\right] \tag{5}$$

Since some population of N_2 does not fully decay in the observed time range we set $k_{23} \approx 0$ for Cystine and GSSG.

$N_3(t)$:

The population $N_3(t)$ of the intermediate vibrationally excited state grows with the rates k_{21} and k_{22} and consists of two sub-ensembles, N_{31} and N_{32}, that decay with rate k_3 respectively. The yields of these populations are ϕ_{31} and ϕ_{32} with $\phi_{32} = 1 - \phi_{31}$:

$$N_3(t) = \textstyle\sum_{j=1}^{2} \sum_{i=1}^{3} \phi_{3j}\, \phi_{2i} N_{3j}^{2i}(t), \text{ where} \tag{6}$$

$$N_{3\mathrm{j}}^{2\mathrm{i}}(t) = k_1 k_{2\mathrm{i}} \left[\frac{e_1}{(k_{2\mathrm{i}}-k_1)(k_3-k_1)} + \frac{e_{2\mathrm{i}}}{(k_1-k_{2\mathrm{i}})(k_3-k_{2\mathrm{i}})} + \frac{e_3}{(k_1-k_{3\mathrm{j}})(k_{2\mathrm{i}}-k_3)}\right] \tag{7}$$

Note that for $k_{23} \longrightarrow 0$ it follows that $N_{3\mathrm{j}}^{23}(t) \longrightarrow 0$.

$N_4(t)$:

The population $N_4(t)$ of the perthiyl radical grows with the rate k_3 and consists of two sub-ensembles, N_{41} and N_{42}, that decay with the respective rates k_{41} and k_{42}. The yields of these populations are ϕ_{41} and ϕ_{42} with $\phi_{42} = 1 - \phi_{41}$:

$$N_4(t) = \sum_{j=1}^{2} \sum_{i=1}^{3} \phi_{4j}\, \phi_{32} \phi_{2i} N_{4j}^{2i}(t) \text{ , where} \tag{8}$$

$$N_{4j}^{2i}(t) = k_1 k_{2i} k_3 \left[\frac{e_1}{(k_{2i}-k_1)(k_3-k_1)(k_{4j}-k_1)} + \frac{e_{2i}}{(k_1-k_{2i})(k_3-k_{2i})(k_{4j}-k_{2i})} + \frac{e_3}{(k_1-k_3)(k_{2i}-k_3)(k_{4j}-k_{32})} + \frac{e_{4j}}{(k_1-k_{4j})(k_{2i}-k_{4j})(k_3-k_{4j})}\right] \tag{9}$$

As before, it is $N_{4j}^{23}(t) \longrightarrow 0$ for $k_{23} \longrightarrow 0$. Moreover, the population of N_4 does not fully decay over the observed time range for Cystine and we set $k_{42} \approx 0$.

For DMDS, we define $k_{41} = (4500 \text{ ps})^{-1}$ according to reference 39 in the main manuscript. For GSSG, the measured time delay range is limited to 100 ps and no decay of the secondary product – attributed to glutathionyl perthiyl radicals – can be observed. We therefore set $k_{41} \approx 0$ for GSSG. Accordingly, we set $\phi_{41} = 1$ for both, DMDS and GSSG (yielding $\phi_{42} = 0$ and making k_{42} irrelevant).

$N_0(t)$:

Finally, the population $N_0(t)$ of the ground state recovers with the rates k_3 and k_{41}:

$$N_0(t) = \sum_{i=1}^{3} \phi_{31} \phi_{2i} N_{03}^{2i}(t) + \sum_{j=1}^{2} \sum_{i=1}^{3} \phi_{4j}\, \phi_{32} \phi_{2i} N_{04j}^{2i}(t) \tag{10}$$

Here, $N_{03}^{2i}(t)$ is defined by

$$N_{03}^{2i}(t) = k_1 k_{2i} k_{31} \left[\frac{\mathrm{Err}}{k_1 k_{2i} k_3} - \frac{e_1}{k_1(k_{2i}-k_1)(k_3-k_1)} - \frac{e_{2i}}{k_{2i}(k_1-k_{2i})(k_3-k_{2i})} - \frac{e_3}{k_3(k_1-k_3)(k_{2i}-k_3)}\right] \tag{11}$$

where, as before, $N_{03}^{23}(t) \longrightarrow 0$ for $k_{23} \longrightarrow 0$.

Further, $N_{04j}^{2i}(t)$ is defined by

$$N_{04j}^{2i}(t) = k_1 k_{2i} k_3 k_{4j} \left[\frac{\mathrm{Err}}{k_1 k_{2i} k_3 k_{4j}} - \frac{e_1}{k_1(k_{2i}-k_1)(k_3-k_1)(k_{4j}-k_1)} - \frac{e_{2i}}{k_{2i}(k_1-k_{2i})(k_3-k_{2i})(k_{4j}-k_{2i})} \ldots \right.$$
$$\left. - \frac{e_3}{k_3(k_1-k_3)(k_{2i}-k_3)(k_{4j}-k_3)} - \frac{e_{4j}}{k_{4j}(k_1-k_{4j})(k_{2i}-k_{4j})(k_3-k_{4j})}\right] \tag{12}$$

where $N_{04j}^{23}(t) \longrightarrow 0$ for $k_{23} \longrightarrow 0$ and $N_{042}^{23}(t) \longrightarrow 0$ for $k_{42} \longrightarrow 0$.

This set of equations was fit to the experimental delay scans in Fig. 2, with the signal evolution at 2466.8 eV (DMDS, Cystine) and 2466.7 eV (GSSG) being proportional to N_2, the signal evolution at 2468.2 eV (Cystine, GSSG) and 2467.7 eV (DMDS) being proportional to N_4, and the signal evolution at 2471.8 to 2472.0 eV (DMDS, Cystine, GSSG) being proportional to N_0.

All parameters were fit globally except for the zero delay points (t_0). Since symmetrically breaking the disulfide bond of one Cystine yields two thiyl radicals a factor of two is applied to N_2. In the case of the delay scans at 2468.2 eV and 2467.7 eV (perthiyl, N_4) a spectral contribution of the Lorentzian lineshape at the thiyl position (F) needs to be included. This contribution has a value of 17.31 % N_2, 8.18 % N_2 and 8.13 % N_2 for DMDS, Cystine and GSSG, respectively. The developed rate equation for N_0 describes the population of N_0 from a starting value of $N_0(0) = 0$. Since this is actually a re-population of a de-populated state, the increasing population has to be added to a starting value of -1 multiplied by the error function, or subtracted, if the amplitude A_{N0} has negative values. The optimized parameters are listed in Tables S5 to S7.

$$\Delta A(\text{Thiyl}) = 2 \cdot A_{N2} \cdot N_2 \tag{13}$$

$$\Delta A(\text{Perthiyl}) = A_{N4} \cdot N_4 + F \cdot 2 \cdot A_{N2} \cdot N_2 \tag{14}$$

$$\Delta A(\text{Parent Bleach}) = A_{N0} \cdot (Err - N_0) \tag{15}$$

Optimal fit parameters for DMDS

Table S5: Optimized parameters of the rate-equation model for DMDS. Parameters marked by a star are globally fit to the three delay scans.

Parameter	2466.8 eV	2467.7 eV	2471.8 eV
**t0 / ps**	0.01908 ± 0.01046	0.03044 ± 0.01242	-0.00767 ± 0.06742
**s* / ps**	0.08999 ± 0.0074	0.08999 ± 0.0074	0.08999 ± 0.0074
**t1* / ps**	0.11191 ± 0.0142	0.11191 ± 0.0142	0.11191 ± 0.0142
**t22* / ps**	5.00348 ± 0.16652	5.00348 ± 0.16652	5.00348 ± 0.16652
**t23* / ps**	3254.91108 ± 286.78995	3254.91108 ± 286.78995	3254.91108 ± 286.78995
**t3* / ps**	0.47395 ± 0.1161	0.47395 ± 0.1161	0.47395 ± 0.1161
**t41* / ps**	4500 ± 0	4500 ± 0	4500 ± 0
**phi_22***	0.66181 ± 0.0037	0.66181 ± 0.0037	0.66181 ± 0.0037
**phi_31***	0.4594 ± 0.20514	0.4594 ± 0.20514	0.4594 ± 0.20514
**phi_41***	1 ± 0	1 ± 0	1 ± 0
**A_thiyl (N2)***	0.08603 ± 7.61546E-4	0.08603 ± 7.61546E-4	0.08603 ± 7.61546E-4
**A_perthiyl (N4)***	0.08205 ± 0.03117	0.08205 ± 0.03117	0.08205 ± 0.03117
**A_bleach (N0)***	-0.10526 ± 0.01072	-0.10526 ± 0.01072	-0.10526 ± 0.01072
**f_thiyl**	1 ± 0	0.1731 ± 0	0 ± 0
**f_perthiyl**	0 ± 0	1 ± 0	0 ± 0
**f_bleach**	0 ± 0	0 ± 0	1 ± 0
**Reduced Chi-Sqr***		10.26759	
**R-Square (COD)**	0.994	0.97697	0.72941
**R-Square (COD)***		0.99372	
**Adj. R-Square***		0.9934	

Optimal fit parameters for GSSG

Table S6: Optimized parameters of the rate-equation model for GSSG. Parameters marked by a star are globally fit to the three delay scans.

Parameter	2466.7 eV	2468.2 eV	2471.9 eV
**t0 / ps**	0.00727 ± 0.01244	0.01091 ± 0.0267	0.09492± 0.01542
**s* / ps**	0.06907 ± 0.00665	0.06907 ± 0.00665	0.06907 ± 0.00665
**t1* / ps**	0.05392 ± 0.01681	0.05392 ± 0.01681	0.05392 ± 0.01681
**t21* / ps**	0.45484 ± 0.023	0.45484 ± 0.023	0.45484 ± 0.023
**t22* / ps**	17.46588 ± 2.97499	17.46588 ± 2.97499	17.46588 ± 2.97499
**t3* / ps**	0.43 ± 0.1065	0.43 ± 0.1065	0.43 ± 0.1065
**t41* / ps**	1E6 ± 0	1E6 ± 0	1E6 ± 0
**phi_21***	0.78776 ± 0.00607	0.78776 ± 0.00607	0.78776 ± 0.00607
**phi_22***	0.1316 ± 0.00665	0.1316 ± 0.00665	0.1316 ± 0.00665
**phi_31***	0.66171 ± 0.03484	0.66171 ± 0.03484	0.66171 ± 0.03484
**phi_41***	0 ± 0	0 ± 0	0 ± 0
**A_thiyl (N2)***	0.10925 ± 0.00304	0.10925 ± 0.00304	0.10925 ± 0.00304
**A_perthiyl (N4)***	0.03573 ± 0.0038	0.03573 ± 0.0038	0.03573 ± 0.0038
**A_bleach (N0)***	-0.20283 ± 0.01153	-0.20283 ± 0.01153	-0.20283 ± 0.01153
**f_thiyl**	1 ± 0	0.0813 ± 0	0 ± 0
**f_perthiyl**	0 ± 0	1 ± 0	0 ± 0
**f_bleach**	0 ± 0	0 ± 0	1 ± 0
**Reduced Chi-Sqr***		1.13979	
**R-Square (COD)**	0.9955	0.73592	0.85158
**R-Square (COD)***		0.98701	
**Adj. R-Square***		0.98643	

5. Point omission in cystine data

By omitting (masking) three points, which deviate from the natural behavior of rising edges, from the induced absorption delay scan after UV excitation of Cystine (Fig. S5, bright green points), the fit of the delay scan at 2466.8 eV can be improved (Fig. S5, black fit). In Table S7 the optimal fit parameters *without* considering these points are listed. Taking all measured data points into account results in a fit that is plotted as the green graph in Fig. S5 for which the model parameters are listed in Table S8. The essential difference between the two sets of fit parameters manifests in the thiyl formation time τ_1:

$$\tau_{1,\text{masked}} = (0.0625 \pm 0.02192)\ \text{ps}$$

$$\tau_{1,\text{unmasked}} = (0.14359 \pm 0.02269)\ \text{ps}$$

The value for $\tau_{1,\text{unmasked}}$ has been reported by Ochmann et al.[57] which considered all measured points. Omitting the data points in Fig. S5 leads to a value of τ_1 that is very similar to the value obtained for GSSG. The width of the instrument response function, s_{masked} is slightly longer than s_{unmasked}. The two thiyl radical decay times, τ_{21} and τ_{22} become slightly longer when masking the points.

Optimal fit parameters for cystine *with* omission of data points

Table S7: Optimized parameters of the rate-equation model for Cystine. Parameters marked by a star are globally fit to the three delay scans.

Parameter	2466.8 eV	2468.2 eV	2472.0 eV
**t0 / ps**	0.04152 ± 0.0162	0.04579 ± 0.01949	0.05666 ± 0.0148
**s* / ps**	0.09742 ± 0.00625	0.09742 ± 0.00625	0.09742 ± 0.00625
**t1* / ps**	0.0625 ± 0.02192	0.0625 ± 0.02192	0.0625 ± 0.02192
**t21* / ps**	0.49122 ± 0.02901	0.49122 ± 0.02901	0.49122 ± 0.02901
**t22* / ps**	6.11944 ± 0.5025	6.11944 ± 0.5025	6.11944 ± 0.5025
**t3* / ps**	0.42569 ± 0.0483	0.42569 ± 0.0483	0.42569 ± 0.0483
**t41* / ps**	248.79465 ± 120.70011	248.79465 ± 120.70011	248.79465 ± 120.70011
**phi_21***	0.64686 ± 0.00825	0.64686 ± 0.00825	0.64686 ± 0.00825
**phi_22***	0.20211 ± 0.00728	0.20211 ± 0.00728	0.20211 ± 0.00728
**phi_31***	0.68452 ± 0.03541	0.68452 ± 0.03541	0.68452 ± 0.03541
**phi_41***	0.42462 ± 0.08746	0.42462 ± 0.08746	0.42462 ± 0.08746
**A_thiyl (N2)***	0.10975 ± 0.00273	0.10975 ± 0.00273	0.10975 ± 0.00273
**A_perthiyl (N4)***	0.07262 ± 0.00821	0.07262 ± 0.00821	0.07262 ± 0.00821
**A_bleach (N0)***	-0.14147 ± 0.00522	-0.14147 ± 0.00522	-0.14147 ± 0.00522
**f_thiyl**	1 ± 0	0.0818 ± 0	0 ± 0
**f_perthiyl**	0 ± 0	1 ± 0	0 ± 0
**f_bleach**	0 ± 0	0 ± 0	1 ± 0
**Reduced Chi-Sqr***		2.82388	
**R-Square (COD)**	0.99654	0.97005	0.91341
**R-Square (COD)***		0.99623	
**Adj. R-Square***		0.99601	

Optimal fit parameters for cystine *without* omission of data points

Table S8: Optimized parameters of the rate-equation model for Cystine. Parameters marked by a star are globally fit to the three delay scans.

Parameter	2466.8 eV	2468.2 eV	2472.0 eV
**t0 / ps**	2.38987E-4 ± 0.00785	7.68162E-5 ± 0.01479	0.05474 ± 0.01638
**s* / ps**	0.08749 ± 0.00586	0.08749 ± 0.00586	0.08749 ± 0.00586
**t1* / ps**	0.14359 ± 0.02269	0.14359 ± 0.02269	0.14359 ± 0.02269
**t21* / ps**	0.4109 ± 0.04565	0.4109 ± 0.04565	0.4109 ± 0.04565
**t22* / ps**	5.67371 ± 0.51574	5.67371 ± 0.51574	5.67371 ± 0.51574
**t3* / ps**	0.45463 ± 0.06286	0.45463 ± 0.06286	0.45463 ± 0.06286
**t41* / ps**	260.33921 ± 154.24387	260.33921 ± 154.24387	260.33921 ± 154.24387
**phi_21***	0.66793 ± 0.01808	0.66793 ± 0.01808	0.66793 ± 0.01808
**phi_22***	0.19296 ± 0.01066	0.19296 ± 0.01066	0.19296 ± 0.01066
**phi_31***	0.65766 ± 0.04104	0.65766 ± 0.04104	0.65766 ± 0.04104
**phi_41***	0.42239 ± 0.10821	0.42239 ± 0.10821	0.42239 ± 0.10821
**A_thiyl (N2)***	0.11991 ± 0.00791	0.11991 ± 0.00791	0.11991 ± 0.00791
**A_perthiyl (N4)***	0.0652 ± 0.00817	0.0652 ± 0.00817	0.0652 ± 0.00817
**A_bleach (N0)***	-0.13882 ± 0.00568	-0.13882 ± 0.00568	-0.13882 ± 0.00568
**f_thiyl**	1 ± 0	0.0818 ± 0	0 ± 0
**f_perthiyl**	0 ± 0	1 ± 0	0 ± 0
**f_bleach**	0 ± 0	0 ± 0	1 ± 0
**Reduced Chi-Sqr***		3.83211	
**R-Square (COD)**	0.99464	0.96932	0.9136
**R-Square (COD)***		0.99531	
**Adj. R-Square***		0.99504	

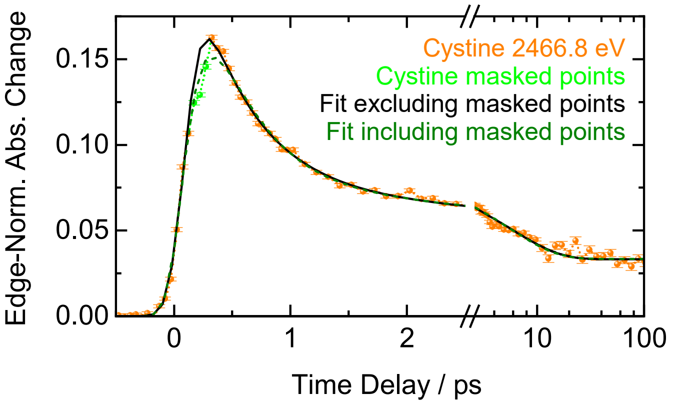

Figure S5. Comparison of Cystine fit curves obtained by global fitting to all Cystine delay scans. The Cystine data (orange) has been taken from a previous publication.[57] A model fit to all data (black curve) is compared to a model fit to all but the three green data points (dark green dashed curve).

6. Orientation of transition dipole moments

Table S9: Angles between transition dipoles and bond directions in L-cystine.

Parent				Thiyl		Perthiyl	
$\angle(CS_1, UV)$	118°	$\angle(CS_1, X_1)$	103°	$\angle(CS_1, X_1)$	87°	$\angle(CS_1, X_1)$	90°
$\angle(CS_2, UV)$	62°	$\angle(CS_1, X_2)$	3°			$\angle(CS_1, X_2)$	89°
$\angle(SS, UV)$	20°	$\angle(CS_2, X_1)$	75°				
$\angle(X_1, UV)$	21°	$\angle(CS_2, X_2)$	91°				
$\angle(X_2, UV)$	117°						

$\angle(CS_1, UV)$ is the angle between the first C-S bond direction and the UV transition dipole moment. Likewise, $\angle(SS, UV)$ is the angle between the S-S bond direction and the UV transition dipole moment. X_1 and X_2 signify the transition dipole moments of the lowest sulfur-1s transitions of the respective compounds. We expect angles in DMDS, GSSG and their photoproducts to be similar.

7. Filtering of measured intensity data

Due to strong fluctuations of intensity and spectral distribution of X-ray pulses from SASE XFELs, we filter the measured intensity values. A correlation plot between the incident X-ray pulse intensity detector (*qbpm*) and the X-ray fluorescence detector (*If*) is shown in Fig. S6 with two filters employed: One filter rejects dark counts (*qbpm* values < 1E-10 detector background for missing X-ray shots) and the other filter rejects the nonlinear response region where *If* values are starting to saturate for high *qbpm* values (*qbpm* values > 4E-9). This upper threshold was determined before the actual energy and delay scans.

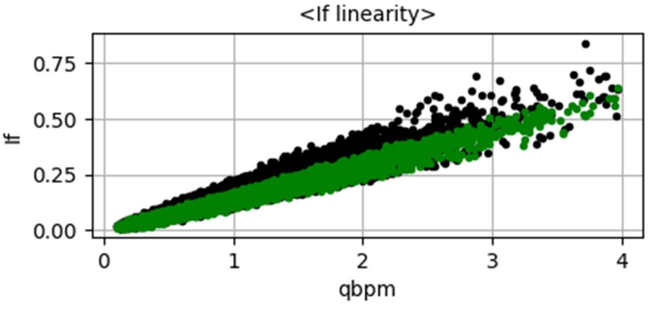

Figure S6. Exemplary correlation plot of measured incident X-ray intensity to X-ray fluorescence intensity with (black) and without (green) UV laser present. For N measured points, for integration of 300 X-ray pulses, 150 points are black and 150 points are green.

The green points in Fig. S6 are measurements without UV excitation, the black points are fluorescence intensity data of the UV-excited sample. The slopes of linear regression fits to both sets of points define the measured signal intensities, the residual between linear fit and measured data points provide the variance and the standard deviation. The difference of the extracted signal intensities defines the absorption change.

For GSSG measurements, the signal of 300 X-ray pulses per point were collected in individual scans, and 300 and 2400 pulses per point were collected for DMDS. Depending on signal quality, up to five individual scans where averaged by a weighted sum. The inverse variance was used as a weight for every intensity data point.

We note that the noise of in the measurements of absorption change vary across the XANES regions because we used total fluorescence yield (TFY) to determine sample absorption and changes thereof: The peak signal at 2470 eV is about 1 V for the unexcited sample. For GSSG, UV excitation decreases this peak TFY intensity by about 6.7% or 60 mV (edge-normalized absorption of GSSG at 2472 eV is 3, the change thereof is 0.2, c.f. Fig. S1) which means that the noise in the two measurements (UV laser ON/OFF) required to determine the absorption change is essentially the same. At 2467 eV the TFY intensity is about 60 meV without UV laser and the UV-induced absorption change amplitude is as large as the bleach signal, i.e. 60 meV. For shot-noise limited measurements we get:

$$S \propto N \ \ , \ \ \Delta S \propto \sqrt{S} \propto \sqrt{N} \ \ , \ \ SNR = S/\Delta S \propto 1/\sqrt{N}$$

With S as the TFY signal intensity for N detected X-ray photons (predominantly sulfur K_α emission), ΔS as the standard deviation of the photon intensity distribution, and *SNR* as the signa-to-noise ratio. This means that ΔS varies with the square root of the TFY intensity S, which in turn is in good approximation proportional to the absorption of the sample. When absorption is measured in transmission, the sample is ideally chosen to be not too strongly absorbing in pump-probe spectroscopy ($\geq 1/e$) and the number of detected photons is similar for all photon energies. Hence, the noise is similar across a measured spectrum.
